# Analog hardware trojan design and detection in OFDM based wireless cryptographic ICs

**DOI:** 10.1371/journal.pone.0254903

**Published:** 2021-07-29

**Authors:** Liakot Ali, Farshad

**Affiliations:** Department of Information and Communication Technology (ICT), Institute of Information and Communication Technology (IICT), Bangladesh University of Engineering and Technology (BUET), Dhaka, Bangladesh; Lanzhou University of Technology, CHINA

## Abstract

Due to Hardware Trojan (HT), trustworthiness of Integrated Circuit (IC) supply chain is a burning issue in Semiconductor Industry nowadays. Over the last decade, extensive research has been carried on HT detection methods for digital circuits. However, the HT issue remains largely unexplored in the domain of Analog Mixed Signal (AMS)/ RF circuit where it is now an appealing target for the attackers. The increasing popularity of Orthogonal Frequency Division Multiplexing (OFDM) based wireless cryptographic ICs in modern communication systems makes it a lucrative target for HT-based attacks which could have a devastating impact on data security. This paper presents a trigger-based Hardware Trojan Threat model that exploits the extended cyclic prefix (ECP) property of the OFDM communication scheme to leak the secret encryption key over low noise Additive White Gaussian Channel (AWGN) and developed a Cyclic Prefix (CP) checker based detection mechanism named “SENTRY” to detect such trojans once it is triggered.

## Introduction

Integrated circuits (ICs) are a core component of all electronic devices and products that are widely used today in our daily life as well as in modern civilization like consumer products, communication, energy, defense, finance, transport, etc. To reduce manufacturing cost and time to market, various part of IC design cycle has been outsourced to different parts of the world [1–3]. However, this globalization of the IC supply chain has resulted in tremendous vulnerabilities and security concerns for mission-critical and safety-critical applications [4–7] and this is known as hardware Trojan (HT). HT is the intentional illegal modification into IC for malicious purposes such as theft of sensitive data, degrade performance and cease operation once HT is triggered [7–9]. Devastating impacts and effects of HT on national security have already been mentioned in the literature [10–11] such as failure of the radar system, problem in navigation and flight control in a Boeing 787, presence of HT in Dell server motherboards deployed in critical applications of US defense, etc. It is found that in the past decade extensive researches from industry, academia, and Govt have been conducted on understanding the different aspects of HT [12–14] and its detection [15–18] and Prevention [19, 20] in digital circuits however the topic remains largely unexplored and missing in the analog/RF ICs [21–25]. It is because, it is very difficult and challenging to design HTs of stealthy nature, small footprint, and indistinguishable nature for analog circuits [8]. Since analog/RF functionalities (wireless communications, sensor actuators, etc.) are being widely used in the contemporary system of this day, it is very important and urgent to conduct a similar study with HTs in vulnerabilities of analog/RF circuits. Due to this urgency, recently many researches are reported in this domain for the last three years. Literature [26] experimentally demonstrated the effectiveness of the HT in an RF front end of a WLAN transceiver and an Adaptive channel estimation-based defense was proposed. Literature [27–30] conducted many similar studies with HTs in the wireless cryptographic ICs and statistical side-channel (power, delay, temperature, current) was proposed for the defense. Some other notable techniques for the study of HT in the wireless cryptographic ICs proposed by researchers are Forward Error Correction (FEC) technique [31], RF Transmission below noise level [32], Hardware dithering technique [33] Information Flow Tracking (IFT) technique [34], self-referencing technique [35–36], etc. The gap in the literature mentioned for the HT in the analog/RF domain so far is that the study is specific to AM/FM modulation scheme which is not very widely used in higher speed wireless communication. Most modern wireless communication system nowadays uses OFDM based communication scheme. Moreover, always on Trojan has been used in the previous research. The issues related to trigger-based Trojan and OFDM-based communication in AMS/RF IC are still unexplored yet. This paper presents research aiming to bridge the said gap by introducing a Time-Triggered Other Payload-based Mixed Signal HT which steals the secret key of a secure Wireless Cryptographic IC exploiting the Extended Cyclic Prefix (ECP) property of the OFDM communication scheme. An earlier version of this research has been presented at the conference [37]. A detailed and matured version of the research will be presented here as per the following organization:

1. Section 2 explains the concept of the OFDM communication scheme including its Cyclic Prefix property.
2. Section 3 contains details of Wireless Cryptographic IC that is designed and used as an experiment vehicle for demonstrating proposed HT. This section focuses on the Trojan Free and Trojan infested versions of an Advanced Encryption Standard (AES-128) + OFDM scheme-based Wireless Cryptographic IC to demonstrate the capabilities of the Hardware Trojan and the detection mechanism.
3. Section 4 discusses the simulation results and discussion. A comparison between the proposed methodology and prior works is also discussed in section 4.
4. Section 5 summarizes our results and the outcome of this research. It also provides some direction to further research works for the future.

## OFDM communication scheme

**Fig 1:**
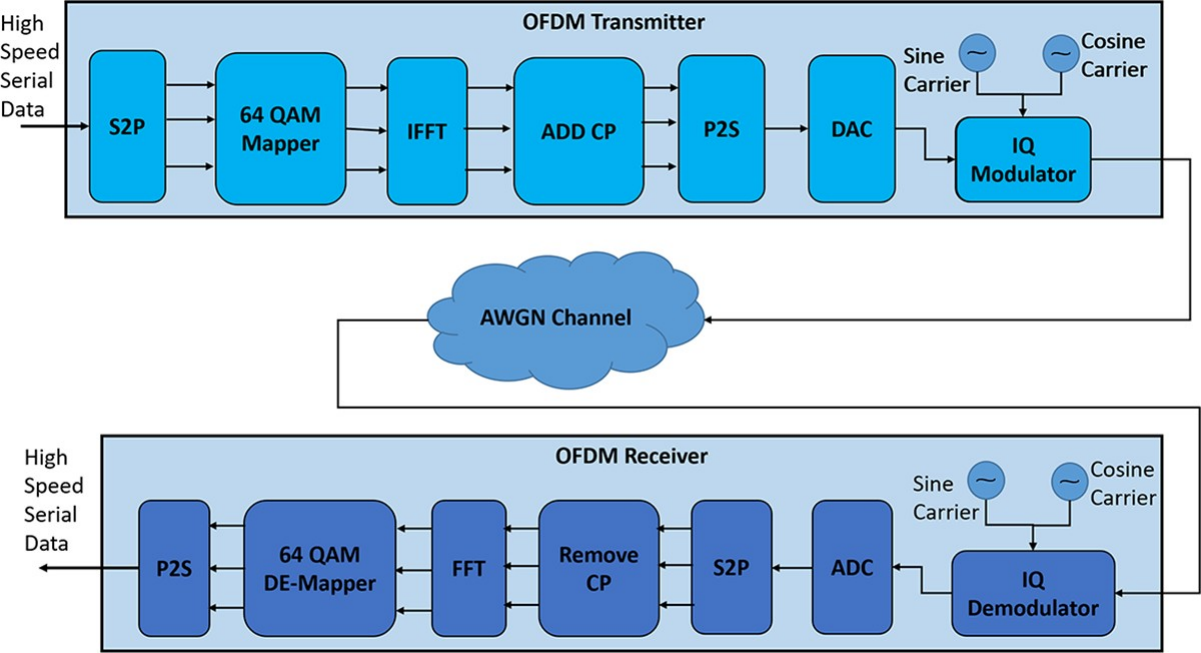
Block diagram of OFDM transmitter and receiver.

OFDM is a multicarrier modulation scheme that is widely used in high-speed communication like 3G, LTE, LTE-Advanced, WiMAX, 5G, etc. OFDM divides the channel bandwidths among multiple orthogonal subcarriers for transmitting the information. For this purpose, high-speed serial data is converted into low-speed parallel data and each data bit is modulated using subcarrier frequency. Fig 1. shows a system-level block diagram of an OFDM Transmitter and Receiver.

The input of the OFDM transmitter block is high-speed serial data which is converted into lower-speed parallel data by using a serial to parallel (S2P) converter. This parallel data is mapped using a data modulator such as Amplitude Shift Keying (ASK), Frequency Shift Keying (FSK), Phase Shift Keying (PSK), Quadrature Amplitude Modulation (QAM), Quadrature Phase Shift Keying (QPSK), etc. The modulated data is fed to an Inverse Fast Fourier Transform (IFFT) block to generate the OFDM Symbol. This operation can be mathematically represented as,

$$F(k\Delta t)=\frac{1}{N}\sum_{n=0}^{N-1}F(n\Delta f)e^{j2\pi nk/N}$$ (1)

Where, F(*k*Δ*t*) = Time-domain component of the signal

N = number of subcarriers

n = order of subcarrier

Δ*f* = Subchannel frequency spacing

The generated OFDM symbol is fed into an add cp block for adding cyclic prefix for reducing the effect to inter symbol interference (ISI) due to multipath propagation. Then parallel data stream is converted into a serial data stream using a parallel to serial converter (P2S). The serial data stream is sent to a digital to analog converter for converting into an analog baseband signal and then passed through a low pass filter (LPF) for removing additional noises. Then the analog baseband data is fed through an IQ modulator which modulates the baseband data higher frequency carrier data and then modulated signal transmitted through an antenna. Mathematically, transmitted OFDM waveform S(t) can be expressed as,

$$S(t)=\frac{1}{N}\sum_{n=0}^{N-1}\left\{A_{n}(t)e^{j(2\pi f_{n}t+\phi_{n}(t)}\right\}$$ (2)

Where,

$$f_{n}=f_{0}+n△f$$ (3)

f_0_ = fundamental frequency

N = total number of subcarrier

n = subcarrier numbers

△*f* = frequency step

A_n_(t) = Amplitude of the n-th carrier

Φ_n_(t) = phase of the n-th carrier

On the receiver end, the received signal is passed through LPF to remove the noise, and then IQ demodulation is performed to separate the baseband data from the carrier. To retrieve the baseband data from the carrier, the IQ modulator and demodulator clocks must be synchronized. To achieve this synchronization pilot symbols are used in the OFDM waveform. Then the analog baseband data is converted into digital using an analog to digital converter (ADC). Then the serial data is converted into parallel data using an S2P converter and after removing the cyclic prefix from the OFDM symbol the data is fed to a Fast Fourier Transform (FFT) block. This demodulates the data from the time domain to the frequency domain. This operation can be mathematically expressed as,

$$F(K)=\sum_{n=0}^{N-1}F(k\Delta t)e^{-j2\pi nk/N}$$ (4)

Where,

F(K) = frequency domain component of the received signal

N = total number of subcarriers

n = subcarrier numbers

*F*(*k*Δ*t*) = time domain component of the received signal

Then the frequency domain data is passed through a QAM demodulator block and then again converted back to serial via a P2S block. Thus, the high-speed data is obtained in the receiver end.

### Cyclic prefix property of OFDM

**Fig 2:**
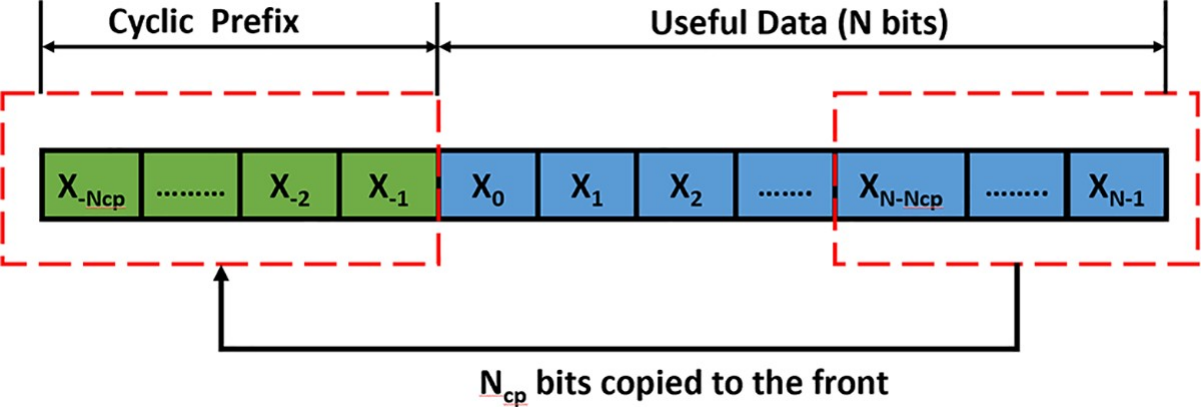
Cyclic prefix property of OFDM communication scheme.

To reduce the effect of inter-symbol interference (ISI) due to multipath propagation OFDM prepends a copy of the end of the OFDM symbol into the beginning of the symbol known as the Cyclic Prefix (CP). CP is always discarded in the receiver end and it is never analyzed. Most used OFDM-based communication schemes like LTE generally use two types of CP; normal and extended [38]. To overcome the inter-symbol interference (ISI) problem, the length of the CP (P) must be greater than or equal to the number of the tap channels (L). In normal CP, the number of tap channels used usually equal to the length of the CP. In the extended cyclic prefix (ECP), the number of tap channels used is greater than the length of the CP. ECP is used to transmit information over an extremely noisy channel. However, if ECP is used in a low noise environment then these extra P-L samples can be used to transmit valid information. Our proposed HT utilizes these extra P-L samples as a covert channel to transmit secret data over the AWGN transmission channel. Fig 2 visually illustrates the cyclic prefix property of the OFDM symbol.

## Hardware trojan threat model implementation and detection

This section contains details of proposed trojan threat model assumptions, the structure of experimental wireless cryptographic IC, proposed Hardware Trojan implementation, and proposed HT Detection Mechanism.

### Threat model

It is necessary to develop an appropriate threat model for the identification and detection of HTs. Literature [9] summarized different threat models to address HT vulnerabilities and potential threats in the IC design cycle as shown in Table 1. Threat model “A” is addressing the possibility of HT insertion via 3^rd^ party IP. In this threat model, the design is developed by trusted design engineers and is fabricated in a trusted foundry. But the design contains many 3^rd^ party IPs which are not produced by a trusted source and thus may contain hidden trojans. The threat model “B” is addressing the possibility of HT injection by a rogue designer or design house. In this model, it is assumed that the design uses a 3^rd^ party IP from a trusted vendor and is fabricated in a trusted foundry, but the design process involves 3^rd^ party contractor design engineers or EDA tools that are untrusted. In threat model “C” the design process is completely outsourced using 3^rd^ party design houses. So, the manufacturer has no control over the design process. So, both the 3^rd^ party IP vendor and design engineers are untrusted. Only the foundry is trusted. The threat model “D” assumes that the design process is trusted but the design is fabricated in an offshore untrusted foundry. In threat model “E” the design process consists of using off-the-shelf IPs which are assembled by offshore 3^rd^ party design houses and fabricated in a secure foundry. In this process, the manufacturer has almost no control over the design process, so both the offshore design house and 3^rd^ party IPs are untrusted only the foundry is trusted. The threat model “F” addresses the issue of HT insertion associated with fabless SoC design house. In this model, the design process is done by in-house design engineers and design usually consists of many 3^rd^ party IPs, and the design is fabricated in an offshore foundry. In this model, the design house has less control over the 3^rd^ party IPs and foundry, so they are considered untrusted and design engineers are trusted.

**Table 1:** Summarized threat model for AMS IC.

HT Threat Model	Description	3PIP Vendor	Design Engineer	Foundry
**A**	Untrusted 3PIP Vendor	Untrusted	Trusted	Trusted
**B**	Untrusted EDA tool or Rouge Developer	Trusted	Untrusted	Trusted
**C**	Untrusted Design House	Untrusted	Untrusted	Trusted
**D**	Untrusted Foundry	Trusted	Trusted	Untrusted
**E**	Commercial off-the-shelf component	Untrusted	Untrusted	Untrusted
**F**	Fabless SoC design house	Untrusted	Trusted	Untrusted

The proposed methodology is developed for Trojan threat model B which states that the design engineer is untrusted and can introduce HTs in the design. This threat model is chosen because due to globalization nowadays it is becoming common for companies to hire 3^rd^ party consultants or design houses during the RTL development process. The proposed adversarial model also assumes that the adversary knows the transmission channel characteristics like channel tap length of the environment where the information theft is planned. It is also assumed that the design is outsourced in many design houses contains many 3^rd^ party IPs and fabricated in the foreign foundry which is a very common case in today’s IC design process. The proposed HT contains two triggers one for the transmitter and one for the receiver. The HT implemented in the transmitter is “sequential triggered other payload type”. The HT implemented in the receiver is a “rare sequence triggered other payload type”.

### Wireless cryptographic IC for hardware trojan threat model

**Fig 3:**
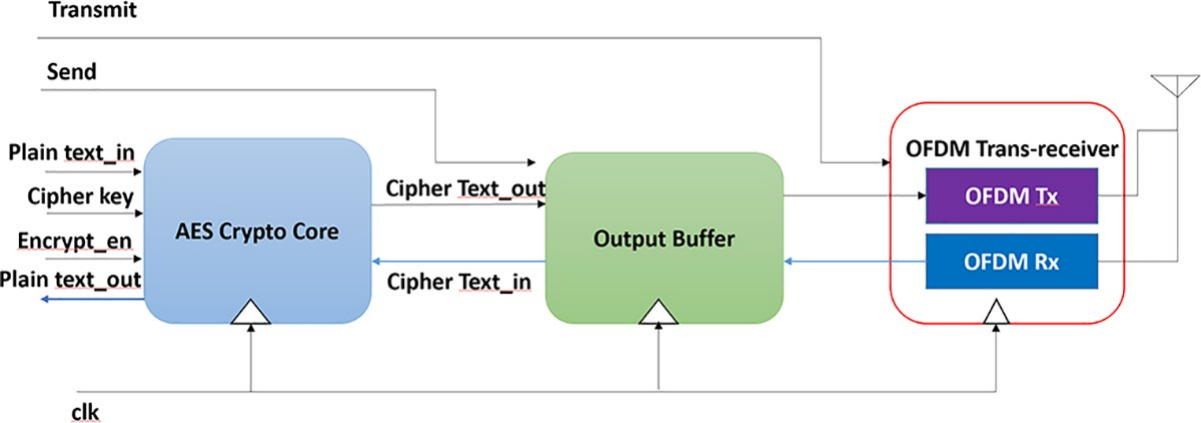
System level block diagram of proposed wireless AES crypto IC.

The experimental vehicle chosen for this paper is an OFDM-based wireless AES cryptographic mixed-signal IC. The digital portion of the IC contains an AES cryptoprocessor and an output buffer. The AES cryptoprocessor can be used for encrypting plain text into secure ciphertext using secure cipher keys or decrypting the ciphertext into plain text using cipher key. The output buffer is used for holding the ciphertext and sending it to the analog domain for transmission or storing the received ciphertext from the analog receiver. The analog portion contains an OFDM trans-receiver for transferring and receiving the secure ciphertext over the Additive White Gaussian Noise (AWGN) transmission channel. A System-level block diagram of the experimental IC chosen for this work is shown in Fig 3.

Two types of experimental IC models are developed for this research work, Trojan Free and Trojan Infested. The Trojan-infested IC acts like Trojan-free IC when HT is dormant. But once the Trojan is triggered it leaks the secret AES encryption key over the AWGN transmission channel by hiding it in the redundant bits (*P-L*) of ECP within OFDM waveform such that it does not distort the waveform. On the transmitter side, the scan chain and Built-In Self-Test (BIST) circuitry are modified such that the Linear Frequency Shift Register (LFSR) circuit in BIST works as a counter that counts clock pulses in regular mode and acts as an LFSR in scan mode. Once the counter reaches a predefined large value then the HT is triggered. The large value of counter required for triggering the HT is chosen such that during post-silicon testing the probability of HT activation is very low. Thus, HT avoids detection during the post-silicon testing stage.

### Trojan free version

**Fig 4:**
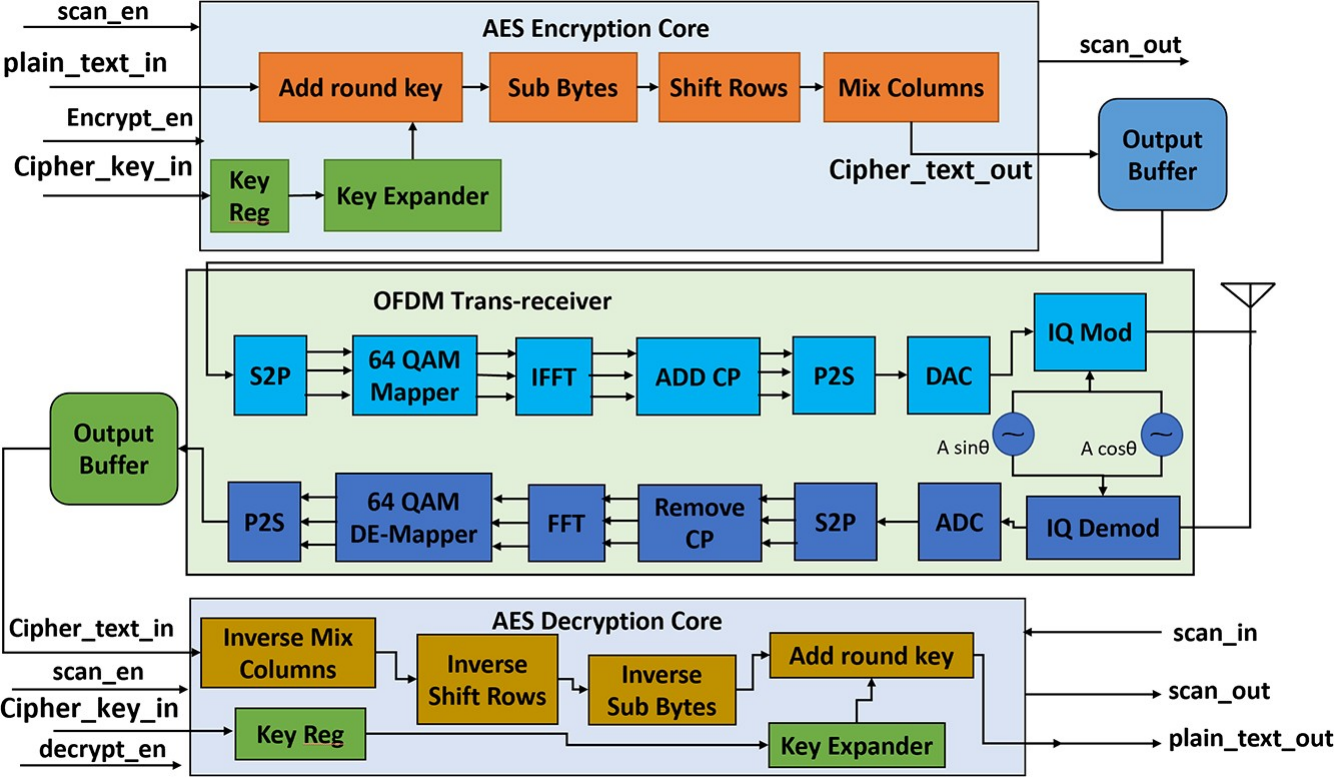
Detailed block diagram of trojan free IC.

The AES crypto core receives a 128-bit plaintext and a 128-bit Cipher key and stores them in on-chip registers. The “plain_text” and “cipher_key” input ports in the design as shown in Fig 4 are used for taking the 128-bit plain text and 128-bit cipher key. The “encrypt_en” must be 1 and “scan_en” must be 0 to enable the AES encryption process. The encryption core implements AES 128 algorithm, so it requires 10 rounds to convert 128-bit plain text into 128-bit ciphertext which is then stored in the output buffer. The ciphertext is then fed into the OFDM trans-receiver module and encrypted data is transmitted into the AWGN channel through the OFDM transmitter.

**Fig 5:**
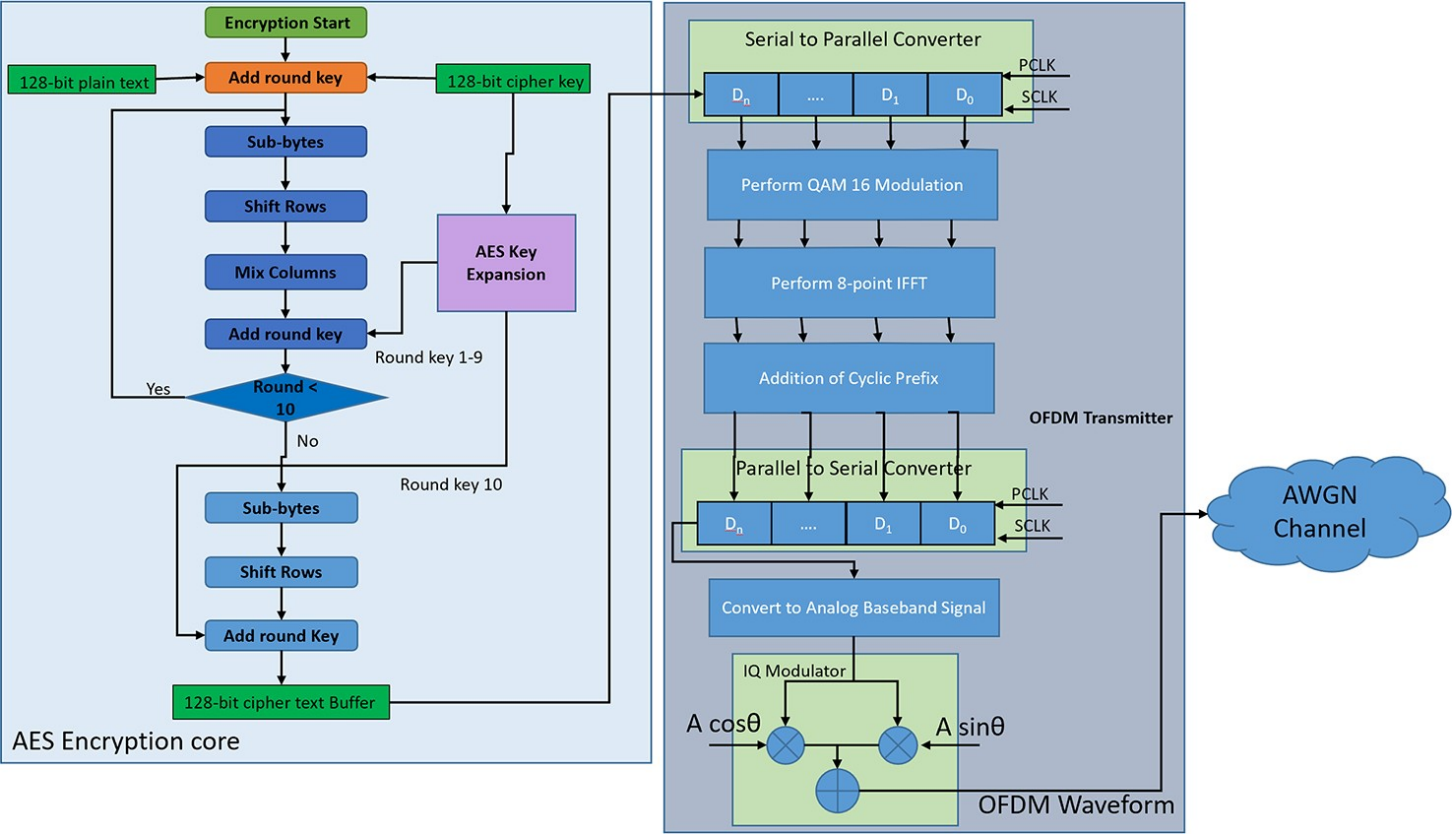
Flow chart of the AES based wireless transmitter module.

OFDM transmitter block consists of serial to parallel block (S2P), 64-point Quadrature Amplitude Modulator (64 QAM mapper), Inverse Fast Fourier Transform (IFFT) block, Cyclic Prefix Addition Block (add_cp), parallel to serial converter (P2S), Digital to Analog Converter (DAC) and IQ modulator. S2P converts the serial data stream coming from AES encryption core output buffer into a parallel data stream which is fed into 64 QAM mappers for constellation mapping. The output of 64 QAM mappers is fed into the IFFT block. IFFT block converts the frequency domain components of OFDM into the time domain components which are fed into the “add_cp” block for adding CP to the OFDM waveform. The output of “add_cp” is again converted into a serial data stream using P2S and then converted into analog signals using a DAC and an IQ modulator. The flow chart of the AES transmitter module is shown in Fig 5.

**Fig 6:**
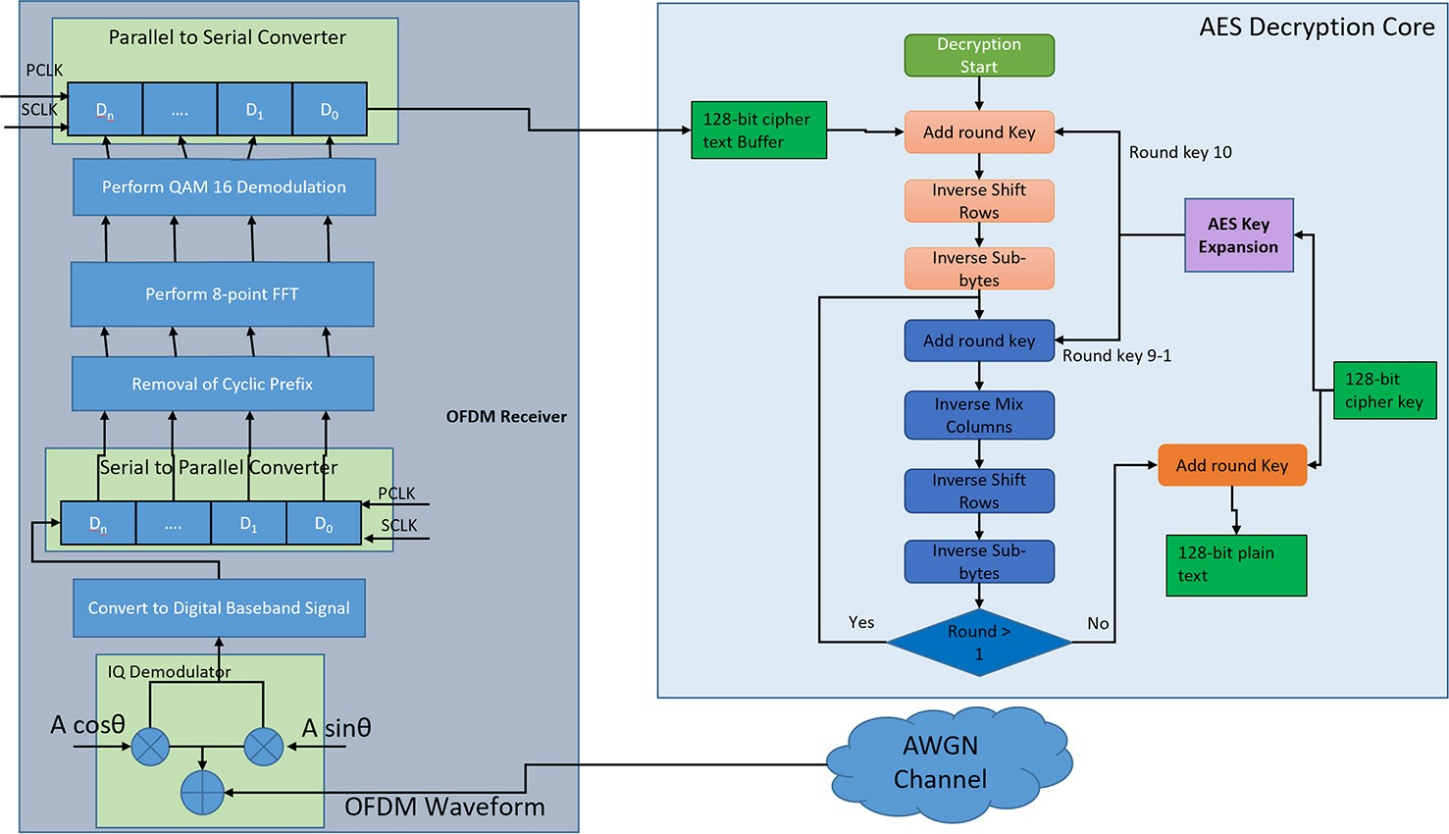
Flowchart of the AES based wireless receiver module.

The receiver block consists of IQ demodulator, Analog to Digital Converter (ADC), S2P, Remove CP (rm_cp) block, Fast Fourier Transform (FFT) block, 64-point QAM de mapper (64 QAM Demod), P2S block. The received IQ modulated data is fed to the IQ demodulator and then to the ADC block. The output of the ADC block is fed to the “rm_cp” block which removes the CP. The output of “rm_cp” is sent into the FFT block which converts the time domain components of the OFDM symbol into frequency domain components. The output of FFT is fed into the 64 QAM Demod block for constellation de-mapping. The output of 64 QAM Demod is converted into a serial data stream using P2S block which is then sent into the output buffer. The AES decrypter core takes the encrypted data from the output buffer and cipher key from the input port “cipher_key_in” and then performs decryption operation. After 10 rounds the decrypted plain text is sent to the output port “plain_text_out”. The detailed flow chart for the receiver operation is shown in Fig 6.

### Trojan infested version

Hardware Trojan is inserted into the design by modifying some circuitry in the digital and analog portions. The transmitter and the receiver contain two different types of trojan triggers. The DFT scan chain and BIST circuitry are modified to create Trojan Triggers in both transmitter and receiver. The following subsection describes the trojan implementation in transmitter and receiver.

### Design of hardware trojan in transmitter

**Fig 7:**
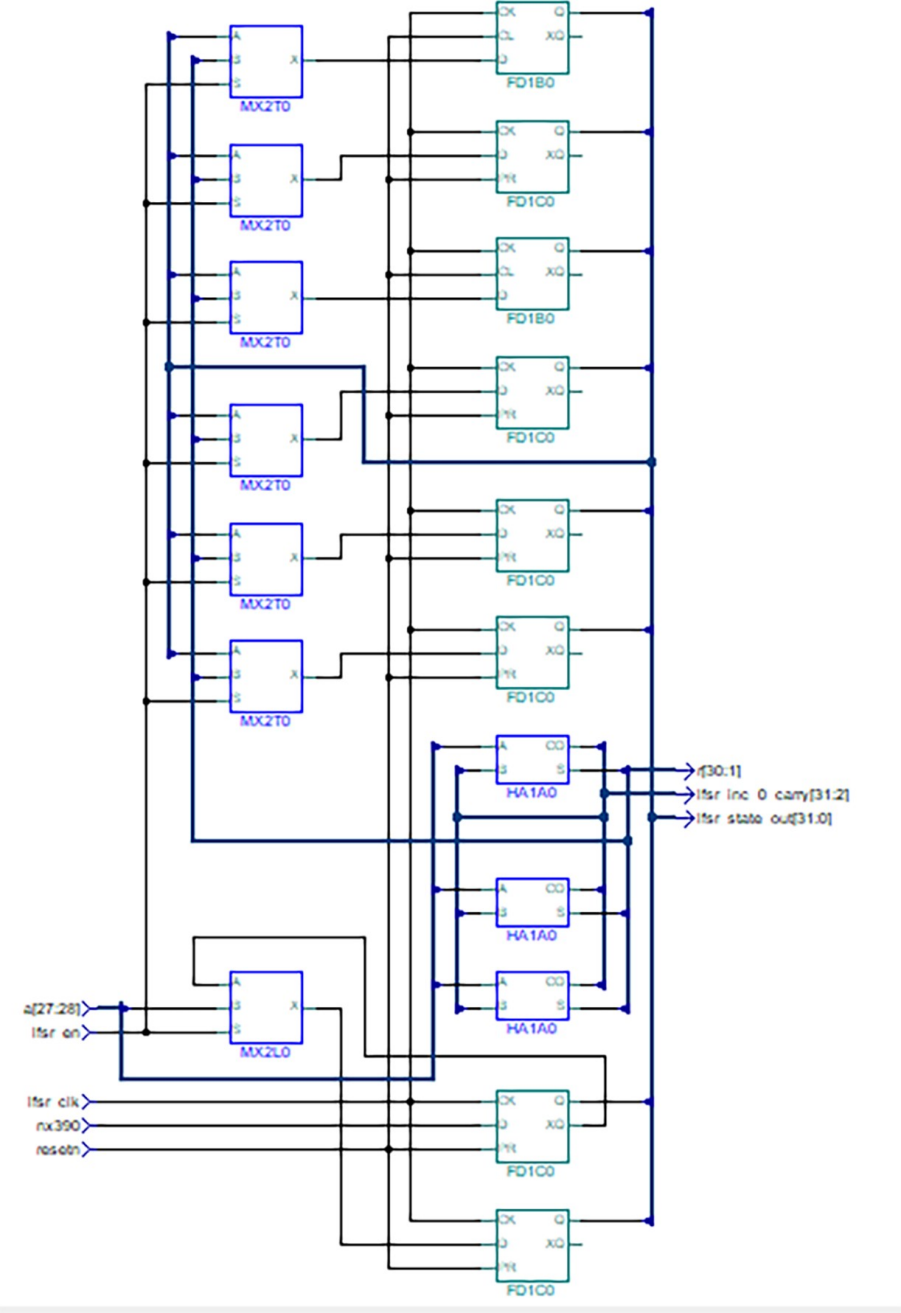
Trojan trigger implementation by modifying LFSR generated using Leonardo spectrum.

In the AES Encrypter module, the LFSR circuitry inside BIST is modified such that it works as LFSR during scan mode and as a clock counter in normal mode. The value of the counter is incremented by one at every positive edge of the clock which acts as a sequential trojan trigger. The modified LFSR schematic is shown in Fig 7. Leonardo Spectrum tool from Mentor Graphics is used to generate the schematic from HDL code. The HT is triggered once the clock counter reaches a predefined large threshold value. In the proposed design, 32’hFFFF_FFFF value has been used as Trojan trigger threshold value to ensure that Trojan remains dormant during post-silicon testing. Once the counter reaches the threshold it sets a flag named “aes_trojan_en” that acts as a trojan trigger signal for the payload. Once set, the flag retains its value until a reset is triggered.

The payload of the HT is designed by adding additional rogue 64 QAM modulator and rogue 8-point IFFT blocks into the transmitter design. The rogue 64 QAM modulator is used to modulate the Trojan data which is fed into the rogue M point IFFT block for generating rogue OFDM symbol. This rogue OFDM symbol is fed into the “add_cp” module through scan inputs for embedding into the ECP of the OFDM symbol. The rogue 64 QAM modulator and the rogue 8-point IFFT block are power gated when the Trojan is dormant and only turns on when the Trojan is activated.

**Fig 8:**
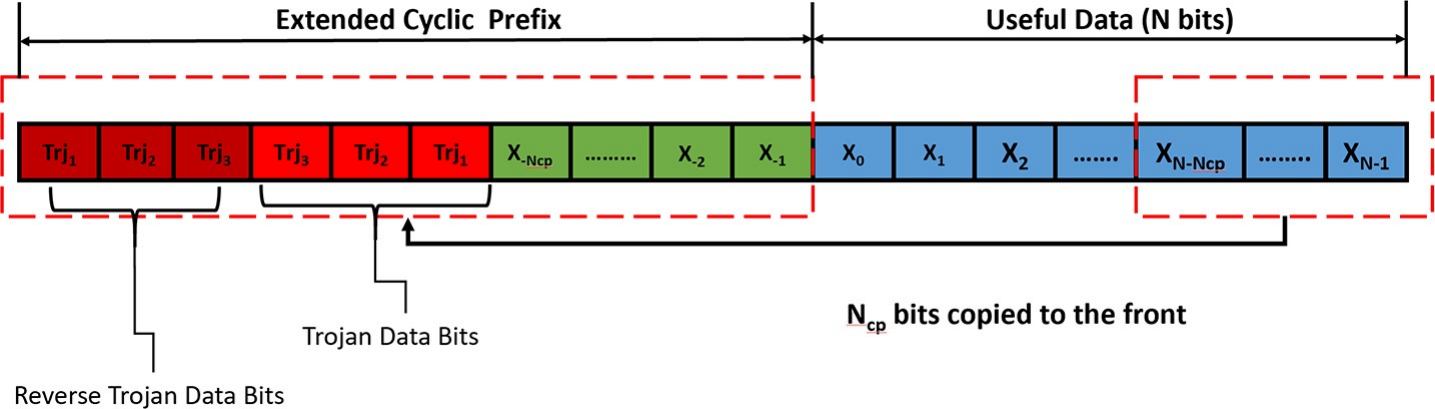
Trojan data insertion in ECP of an OFDM symbol.

This mechanism helps the Trojan to avoid detection via side-channel power analysis. Once activated on the digital portion, the HT steals the secret AES encryption key from the “key_reg” register. A modified DFT scan chain is used to store the stolen value and pass it to the rogue 64 QAM modulator for constellation mapping. The mapped output is then fed into the rogue 8-point IFFT block which creates the Trojan OFDM symbol. The output of the IFFT block is then sent into the “add_cp” block through scan inputs to substitute a portion of the valid ECP with the trojan OFDM symbol. It is assumed that the attacker knows the fading channel tap length and the length of fading channel (L) is less than or equal to half of CP length (P) i.e. L≤ P/2. So, the first P/2 samples of ECP retain their original data, the next P/4 samples are replaced by the Trojan OFDM symbol and the last P/4 locations contain the repeated version of the Trojan OFDM symbol. Thus, creating the contaminated OFDM transmitter waveform. Fig 8 illustrates the Trojan OFDM symbol embedding process inside a valid OFDM symbol.

**Fig 9:**
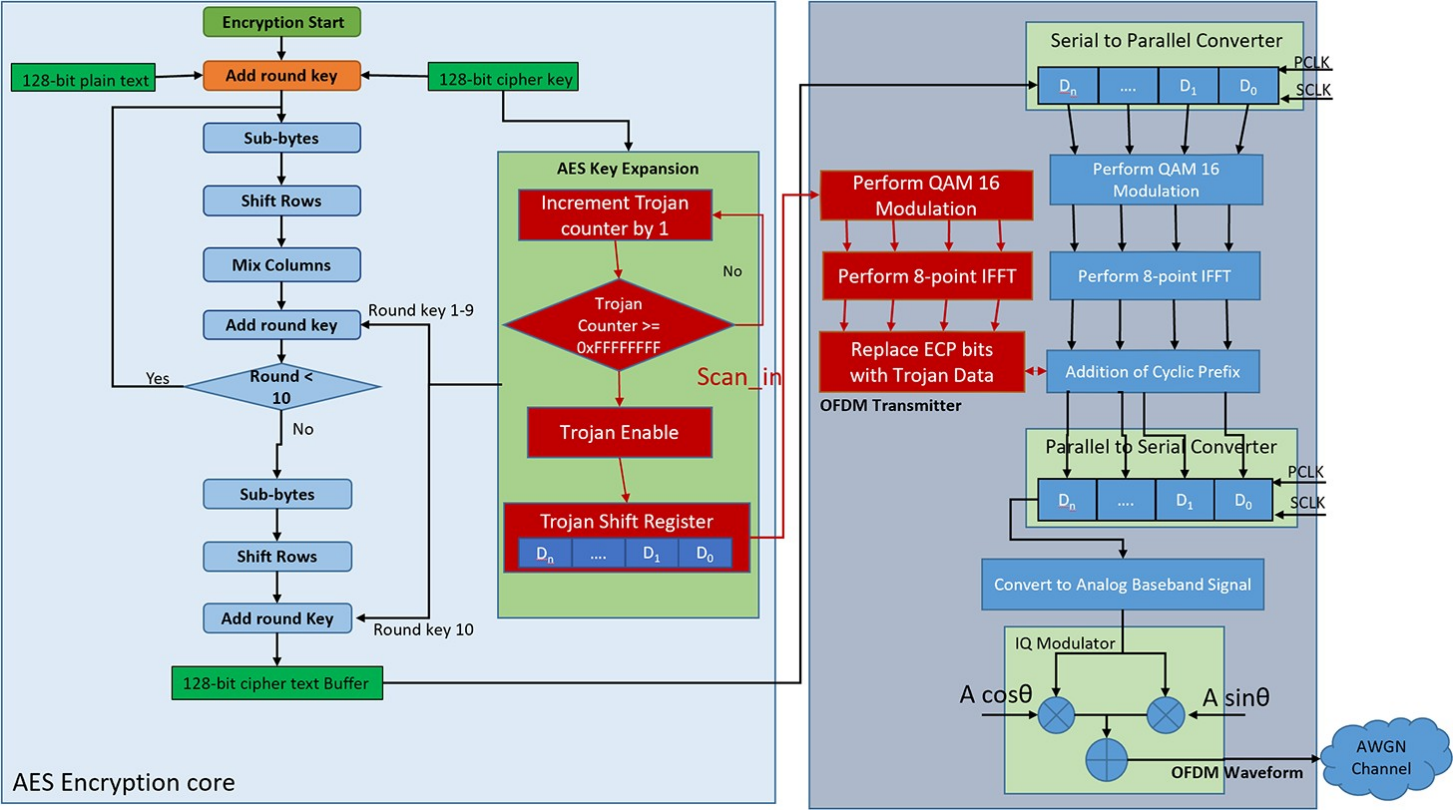
Flowchart of HT infested wireless transmitter module.

The detailed flow diagram of the HT infested transmitter module is shown in Fig 9 where HT-related blocks are marked in red.

### Design of hardware trojan in receiver

**Fig 10:**
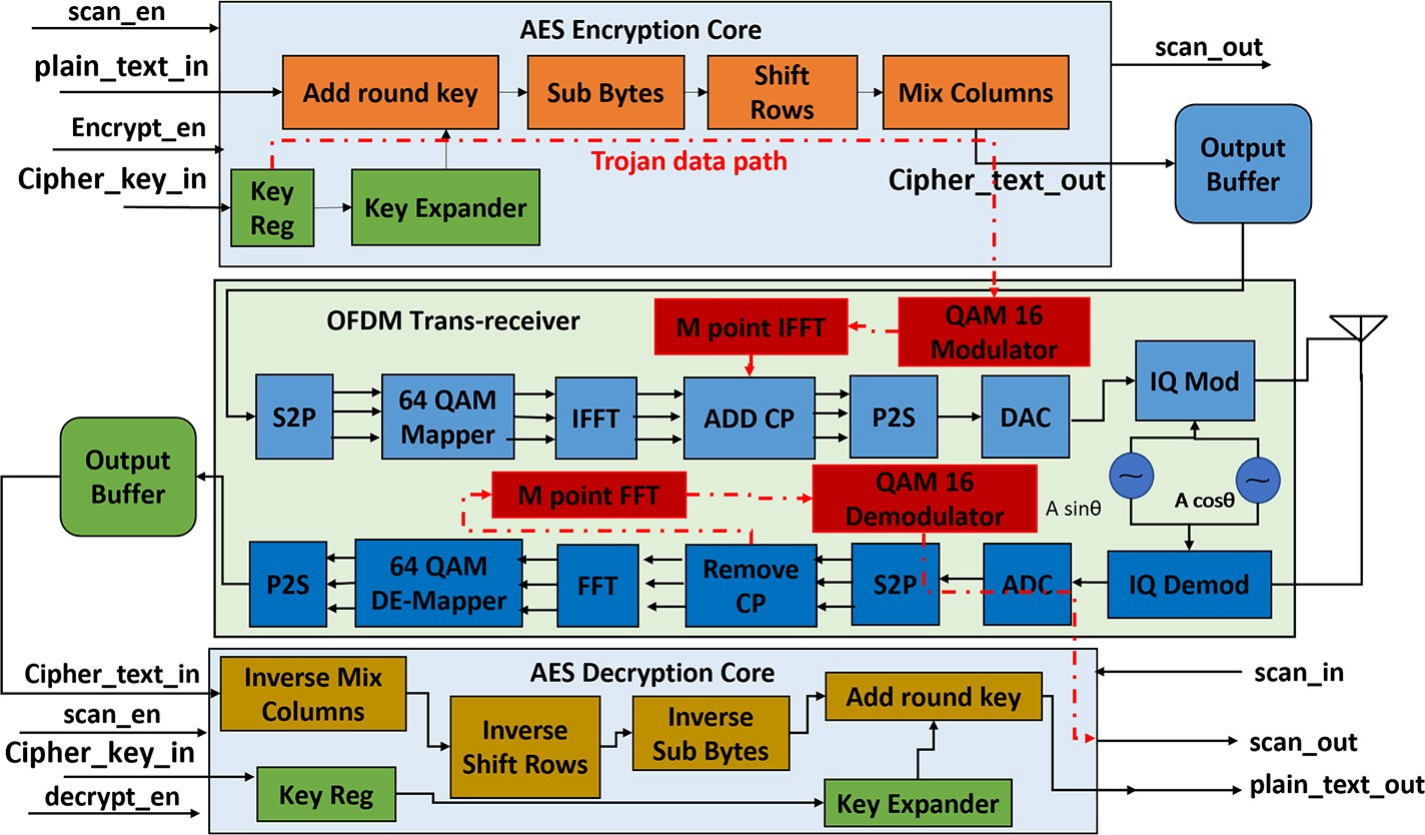
Detailed block diagram of trojan infested IC.

On the receiving side, the HT is enabled by using a rare combination of input ports. In this experiment scan_in [0] [0] = 1 and scan_en = 0 sequence of the scan ports is chosen as Trojan trigger sequence. This sequence is chosen because during normal operation i.e. scan_en = 0 no input is driven into the scan ports. So, it is very unlikely to hit this sequence during the test environment. In the receiver, the trojan payload is implemented by adding an additional rogue 64 QAM demodulator and rogue 8-point FFT which is power gated once the trojan is deactivated. Once the HT is enabled P/4 locations of ECP are extracted as rogue data symbol which is then fed to the rogue 8-point FFT to convert them from time-domain component to frequency domain component. The output of 8-point FFT is then fed to the rogue 64 QAM demodulator block to perform constellation de-mapping. Thus, secret AES encryption key bits are recovered. Generally, based on the length of ECP the HT distributes the secret cipher key among many OFDM packets. The extracted trojan data is then transferred to the attacker via “scan_out” ports. A detailed block diagram of the proposed Trojan infested IC is shown in Fig 10.

**Fig 11:**
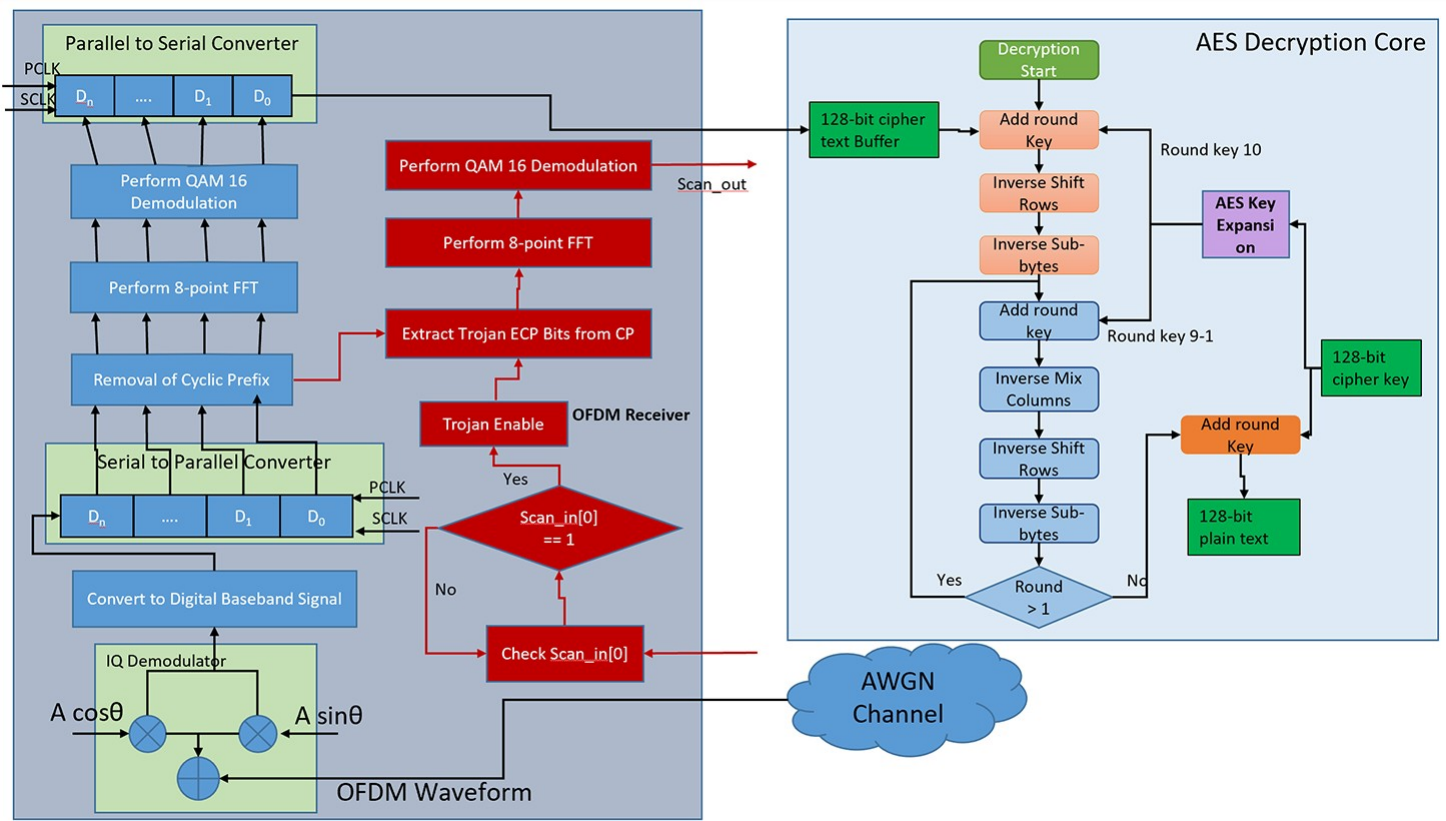
Flowchart of HT infested wireless receiver module.

Fig 11 illustrates the detailed flow diagram of the HT infested receiver module where the HT-related flow is highlighted in red.

### Proposed hardware trojan detection mechanism

The proposed HT relies upon its long-dormant time and rare input activation sequence to evade detection during post-silicon testing. So, side-channel analysis of power and delay are not effective to detect the trojan in the dormant phase. As the receiving side never checks the cyclic prefix so the effect of the trojan is hard to detect from the receiving side. Transmission channel profiling may be a good way to identify the validity of using ECP for transmitting data, but this is not easy to do for every deployment environment and require extensive calculations that are time and power expensive. This paper proposes a cyclic prefix-based hardware trojan detection system named “SENTRY” that works as an OFDM symbol validator on the transmitter side. The SENTRY can be placed inside the transmitter circuit or on-chip. The details of the “SENTRY” implementation are described in the following subsection.

### Design of SENTRY

**Fig 12:**
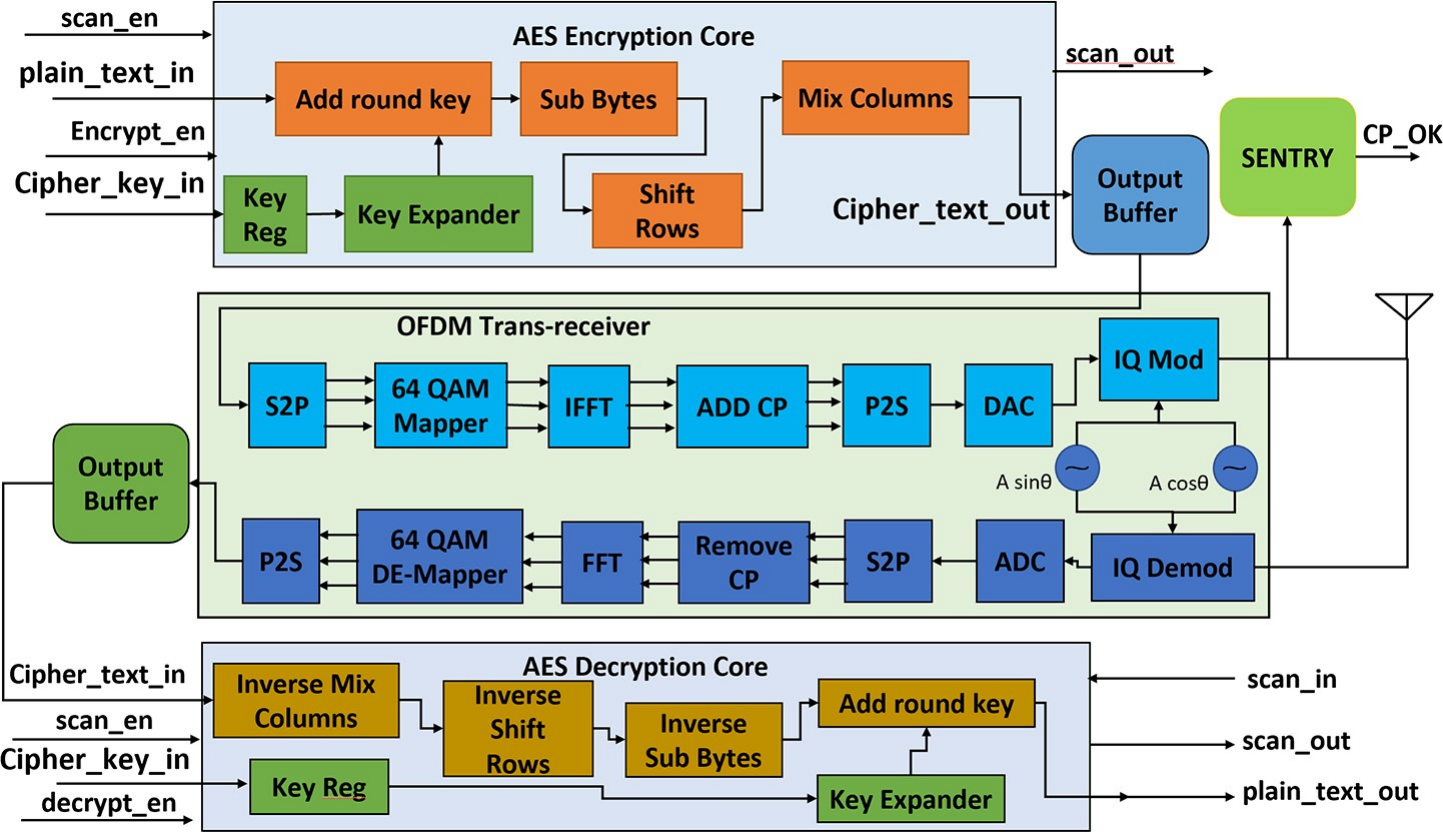
Placement of SENTRY in detailed block diagram of cryptographic IC.

**Fig 13:**
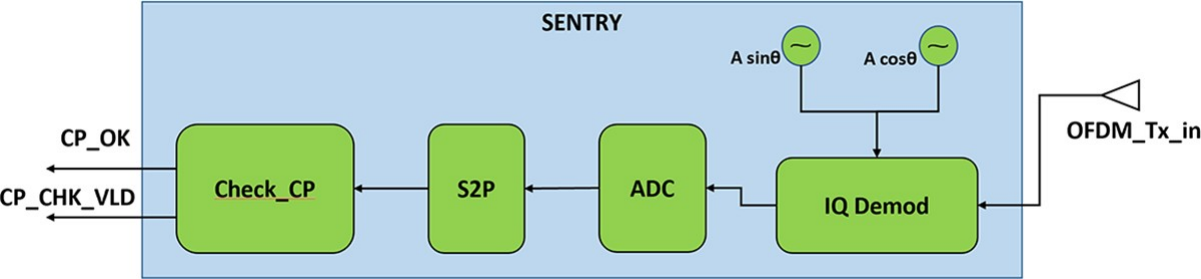
Detailed block diagram of SENTRY.

The SENTRY works as a feature-limited version of the receiver module. The main purpose of the SENTRY is to validate the cyclic prefix of the OFDM symbol. This chip is designed by a separate design team without any interaction with the cryptographic IC design team and is fabricated in a separate foundry. The split fabrication technique can be used if the manufacturer intends to put the SENTRY module inside the transmitter. Then integration of SENTRY into the cryptographic IC must be done in a secure facility and the placement of SENTRY should be very close to the IQ modulator. If SENTRY is placed on-board its placement must be very close to the crypto transmitter. Figs 12 and 13 show the position of the SENTRY in the detailed block diagram and detailed block diagram of SENTRY respectively.

**Fig 14:**
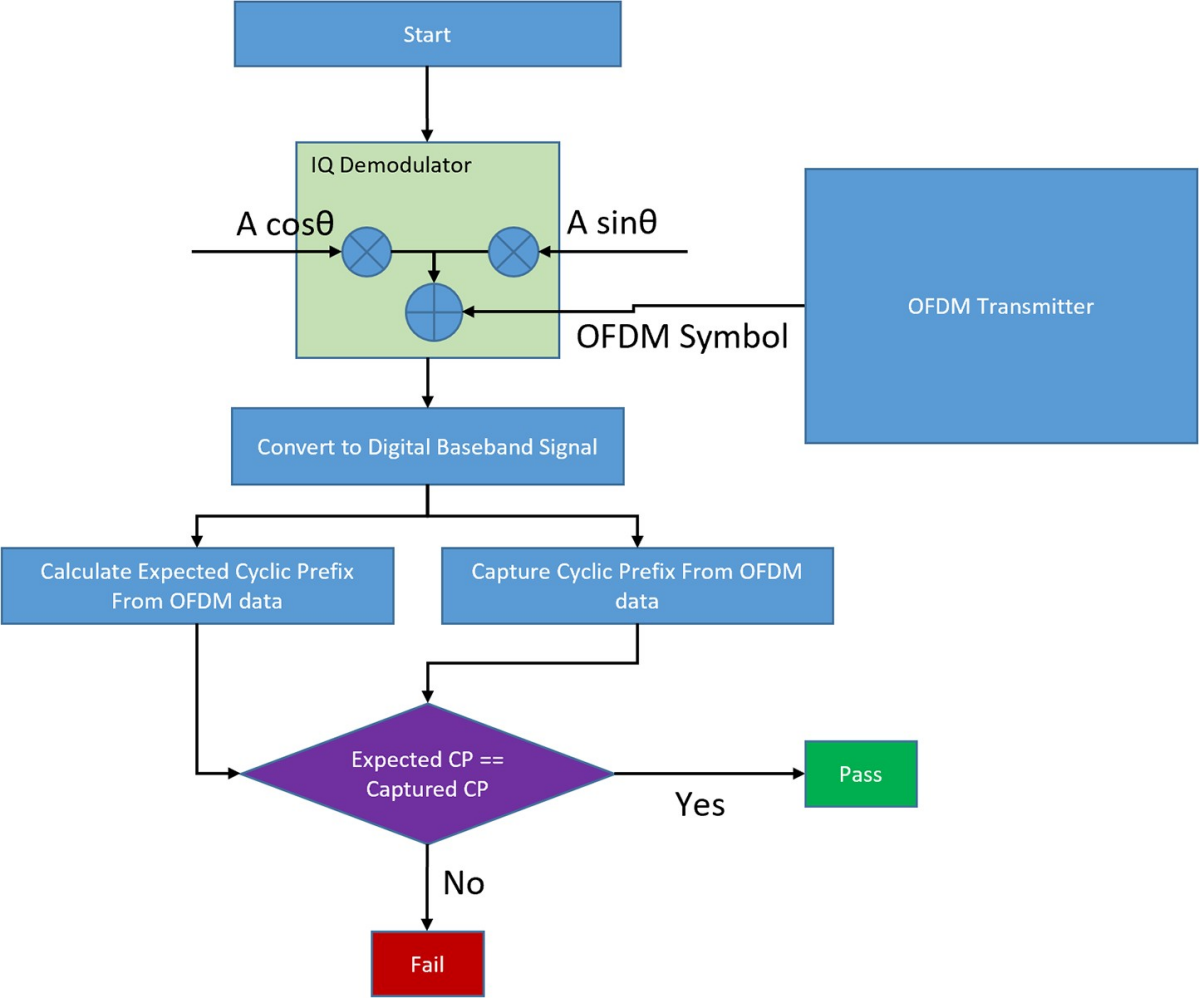
Flowchart of SENTRY.

SENTRY takes in the OFDM symbol from the output of the IQ modulator and then performs IQ demodulation to separate the baseband signal from the high-frequency carrier. Then the analog baseband signal is converted into a digital data stream using an ADC module and then the serial digital data stream is converted into a parallel data stream using a serial to parallel converter (S2P). This parallel data stream is fed to the “Check_CP” module which validates the cyclic prefix. As cyclic prefix (CP) is a repeated form of the end part of the OFDM symbol so, in a trojan-free case cyclic prefix should always match with the corresponding end position data in the OFDM symbol. This comparison is done within a very tight tolerance margin to account for thermal noise and other noise parameters like process variation, internal electromagnetic interference. If the CP matches with data of the respective position, then CP_OK outputs are set high else they are set low. The CP_CHK_VLD signal is set high whenever the comparison is done to indicate a valid CP check. By observing these two signals the sender can identify if the transmitter is operating correctly. Fig 14 shows the detailed flowchart for the SENTRY module.

## Simulation results

Both Trojan-infested and Trojan-free wireless crypto IC design is simulated in Cadence Incisive Unified Simulator (IUS). AWGN transmission channel is used with a very high SNR value for simulating a low noise environment. The following subsection presents the simulation results, findings, and comparison of the proposed HT threat model with existing threat models.

### Experimental setup

The AES-based wireless cryptographic IC is designed using SystemVerilog and simulated using Cadence IUS version 15.2. To demonstrate the proposed threat model, first the trojan free wireless cryptographic IC simulated with a random 128-bit plain text and 128-bit cipher key for generating 128-bit ciphertext using AES cryptoprocessor and transmitting it over AWGN transmission channel using OFDM transmitter. The same cipher key is applied in the receiver to demodulate and decrypt the received noisy OFDM data. The plain text provided to the transmitter and plain text retrieved in the receiver is compared to verify the correct functionality of the design. The effects of the AWGN channel are modeled by creating a behavioral model that generates AWGN noise in the testbench side and it is added to the transmitter output.

Then the trojan-infested IC is simulated with dormant trojan and its behavior is captured and compared with that of the trojan-free device. Then the trojans are triggered in the transmitter and the receiver and behaviors are captured. The “scan_ports” are sampled at the receiver end and the captured data is checked with the randomly chosen 128-bit cipher key. Thus, the data leakage scenario is simulated.

To simulate the detection mechanism, the SENTRY module is connected to the output of the transmitter module before the addition of AWGN noise. Simulation is performed for trojan-free design, trojan-infested design with the dormant trojan, and trojan-infested design with the active trojan.

### Detection evasion mechanism of proposed hardware trojan

**Fig 15:**
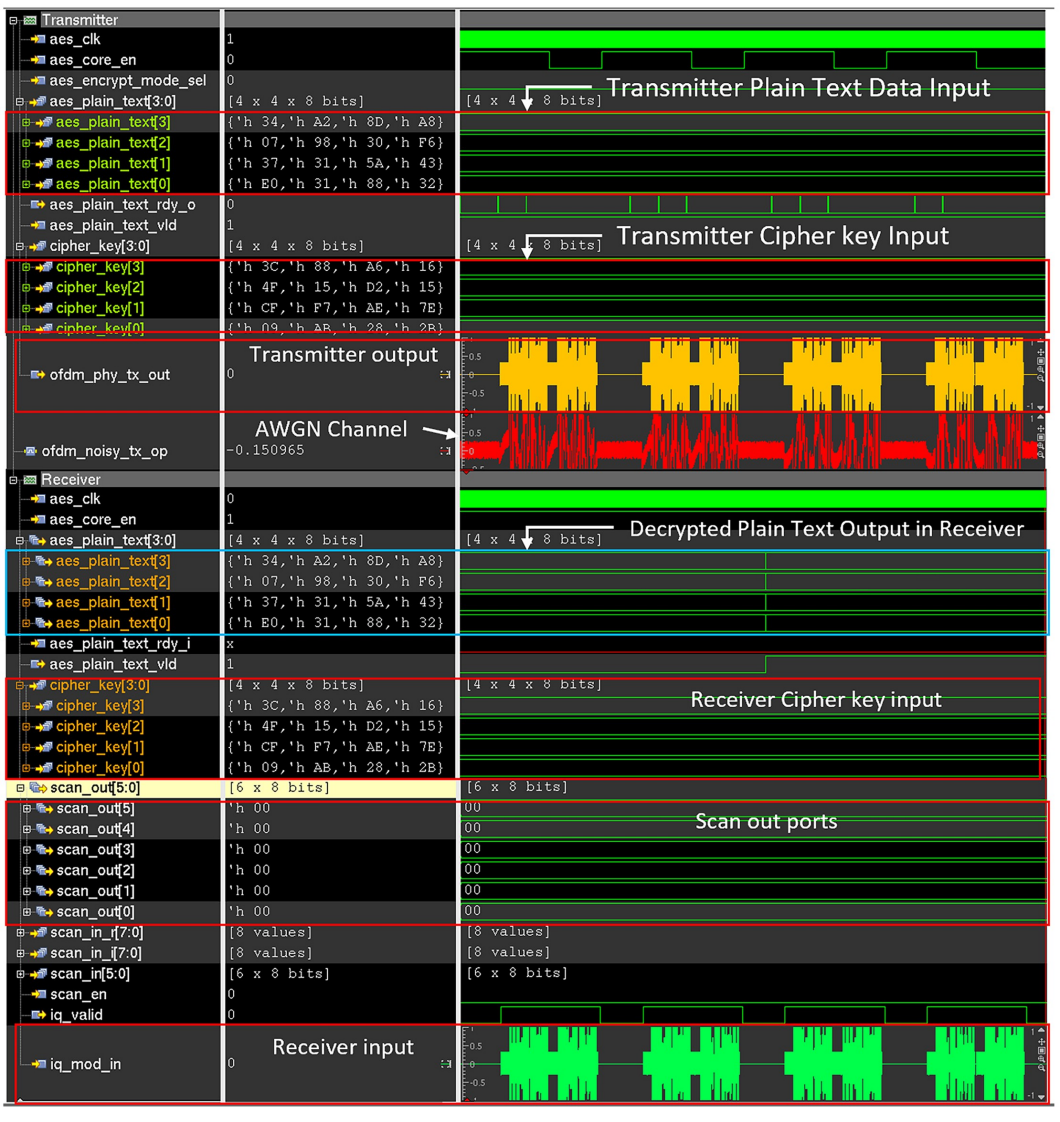
Simulation waveform of trojan free wireless cryptographic IC.

**Fig 16:**
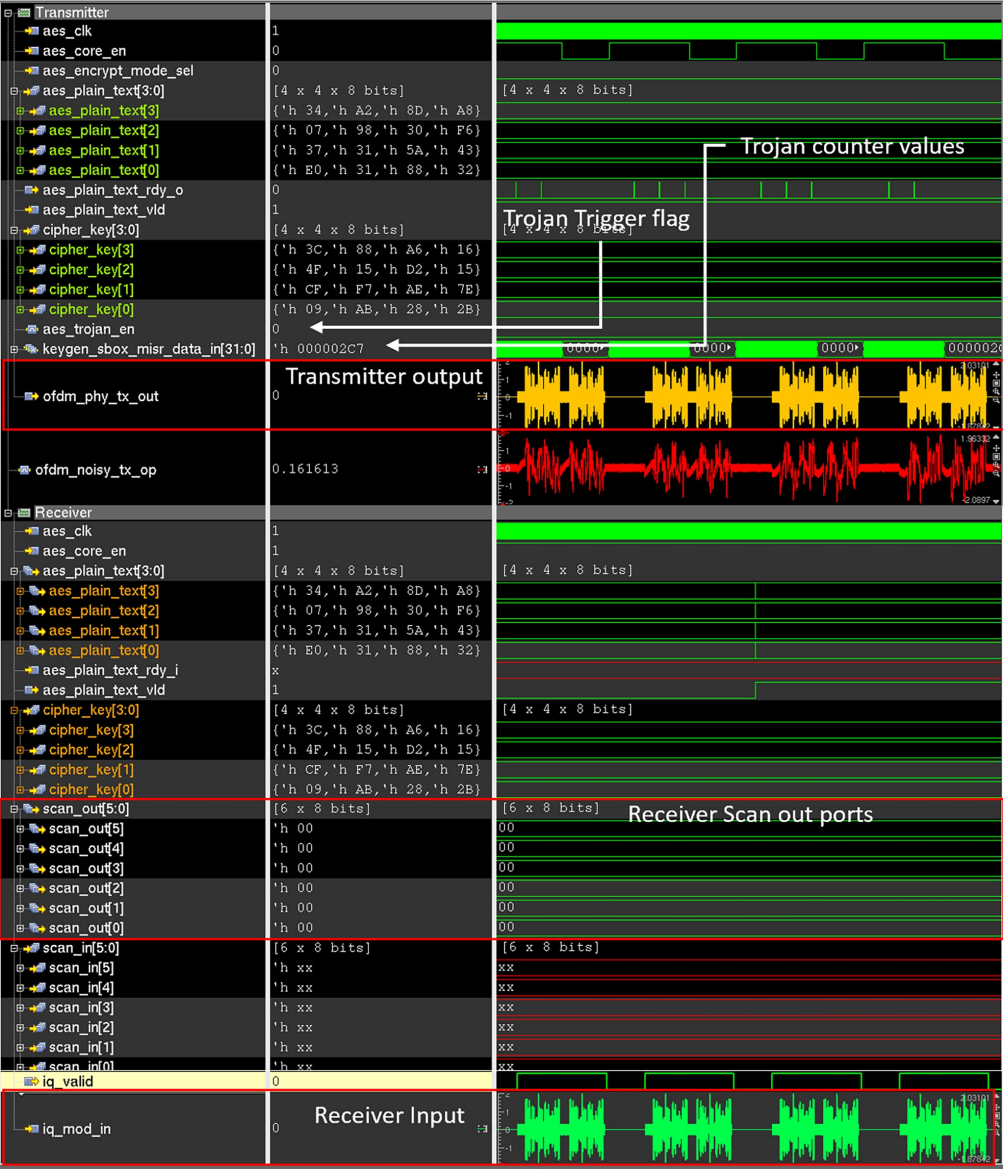
Simulation waveform of trojan infested wireless cryptographic IC when HT is dormant.

**Fig 17:**
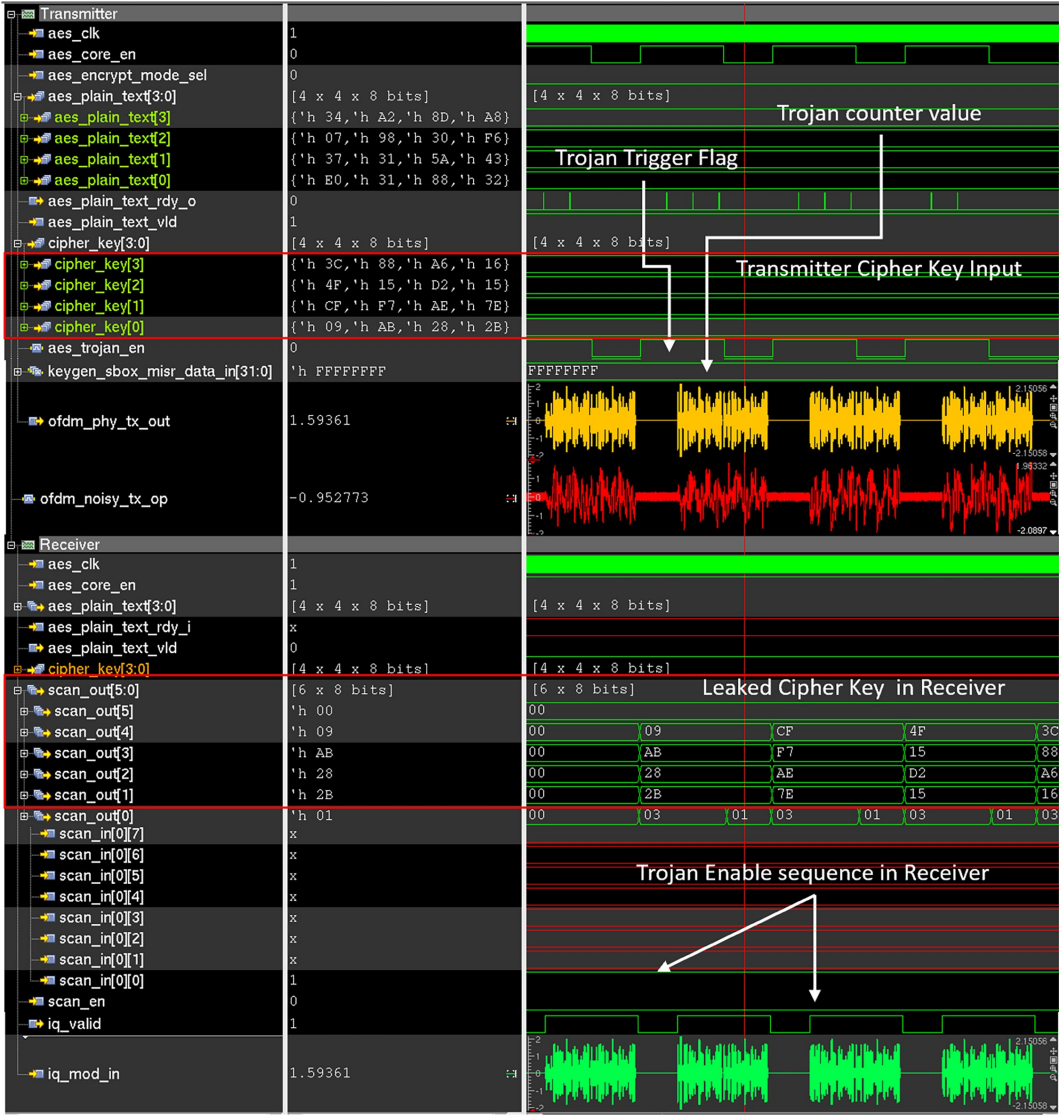
Simulation waveform of the trojan infested wireless cryptographic IC when HT is active.

The proposed HT does not hamper the functionality of the AES crypto core nor does it distort the OFDM waveform to a visually noticeable degree once activated. During the dormant phase, all additional rogue circuitry like 64 QAM Modulator, 64 QAM Demodulator, 8-point IFFT, 8-point FFT is power and clock gated. So, during the dormant stage of HT, the power signature of Trojan-infested IC is identical to that of the Trojan Free IC. The large activation threshold value of the clock counter is required to trigger the trojan in the transmitter and rare input sequence values required to activate the trojan in the receiver increase the probability of the trojan remain dormant during the IC testing phase. Both the trojan-free and the trojan-infested IC are simulated using cadence IUS. To observe the effect of the HT in the dormant stage, both HT free and HT infested transmitter is provided with 128-bit plaintext matrix $\left[\begin{array}{cccc} 0x32 & 0x88 & 0x31 & 0xE0\\ 0x43 & 0x5A & 0x31 & 0x37\\ 0xF6 & 0x30 & 0x98 & 0x07\\ 0xA8 & 0x8D & 0xA2 & 0x34 \end{array}\right]$ and 128-bit cipher key matrix for AES encryption $\left[\begin{array}{cccc} 0x2B & 0x28 & 0xAB & 0x09\\ 0x7E & 0xAE & 0xF7 & 0xCF\\ 0x15 & 0xD2 & 0x15 & 0x4F\\ 0x16 & 0xA6 & 0x88 & 0x3C \end{array}\right]$. Then the necessary control signals are provided to start the encryption and transmission process. The transmitter output port “ofdm_phy_tx_out” in Figs 15 and 16 shows the generated OFDM symbol. The “ofdm_noisy_tx_op” signal in Figs 15 and 16 shows the transmitted OFDM symbol over low noise AWGN channel containing both OFDM symbol and AWGN noise. The “aes_trojan_en” flag shown in Fig 17 indicates the status of the HT trigger. This flag is set to 0 when HT is dormant and set to 1 when HT is triggered. On the receiver side, the same cipher key is provided to perform the decryption operation and decrypted plain text is compared with the plain text provided in the transmitter end. The scan-out ports named “scan_out” in Figs 15 and 16 in the receiver are also observed and no suspicious activity is found. The “iq_mod_in” signal in Figs 15 and 16 shows the received OFDM symbol in the receiver. The simulation waveform of the HT-free IC as shown in Fig 15 is identical to that of HT-infested IC when the HT is dormant as shown in Fig 16. As the trojan only affects the ECP property of the OFDM waveform which is never checked and always discarded in the receiver end so, the legitimate data is unaffected by the trojan activity. As the proposed trojan does not hamper or alter the data payload of the OFDM waveform so, normal users can not be able to detect the presence of the trojan by monitoring the transmission waveform.

### Stealing and leaking the secret data

Once the trojan is activated on the transmitter side, it steals the secret AES encryption key and modifies the ECP bits to embed the trojan data. Fig 17 shows the transmitter and receiver waveform of the Trojan-infested wireless crypto IC when the trojan is activated. In the transmitter waveform shown in Fig 17, the stolen AES cipher key is embedded into the ECP of the OFDM symbol, 16-bit at a time, and transmitted over the AWGN transmission channel. So, the trojan needs at least 8 transfers to completely transmit the stolen 128-bit cipher key.

In the receiver waveform shown in Fig 17, the cipher key is extracted from ECP and sent to the attacker via “scan_out” ports. The experimental setup used in this paper uses scan_out [1:4] for leaking the cipher key to the attacker once the rare input sequence i.e. scan_en = 0 and scan_in [0][0] = 1 is provided in the receiver. scan_out [0][1] is used as an output sample clock, that can be sampled as a valid signal by the attacker during cipher key extraction. The scan_out [0][0] bit indicates that the HT is activated in the receiver end. As shown in Fig 17 the values of the scan_out [1] to scan_out [5] match the 128-bit cipher_key [3:0] provided in the transmitter.

### Simulation result of trojan detection mechanism

**Fig 18:**
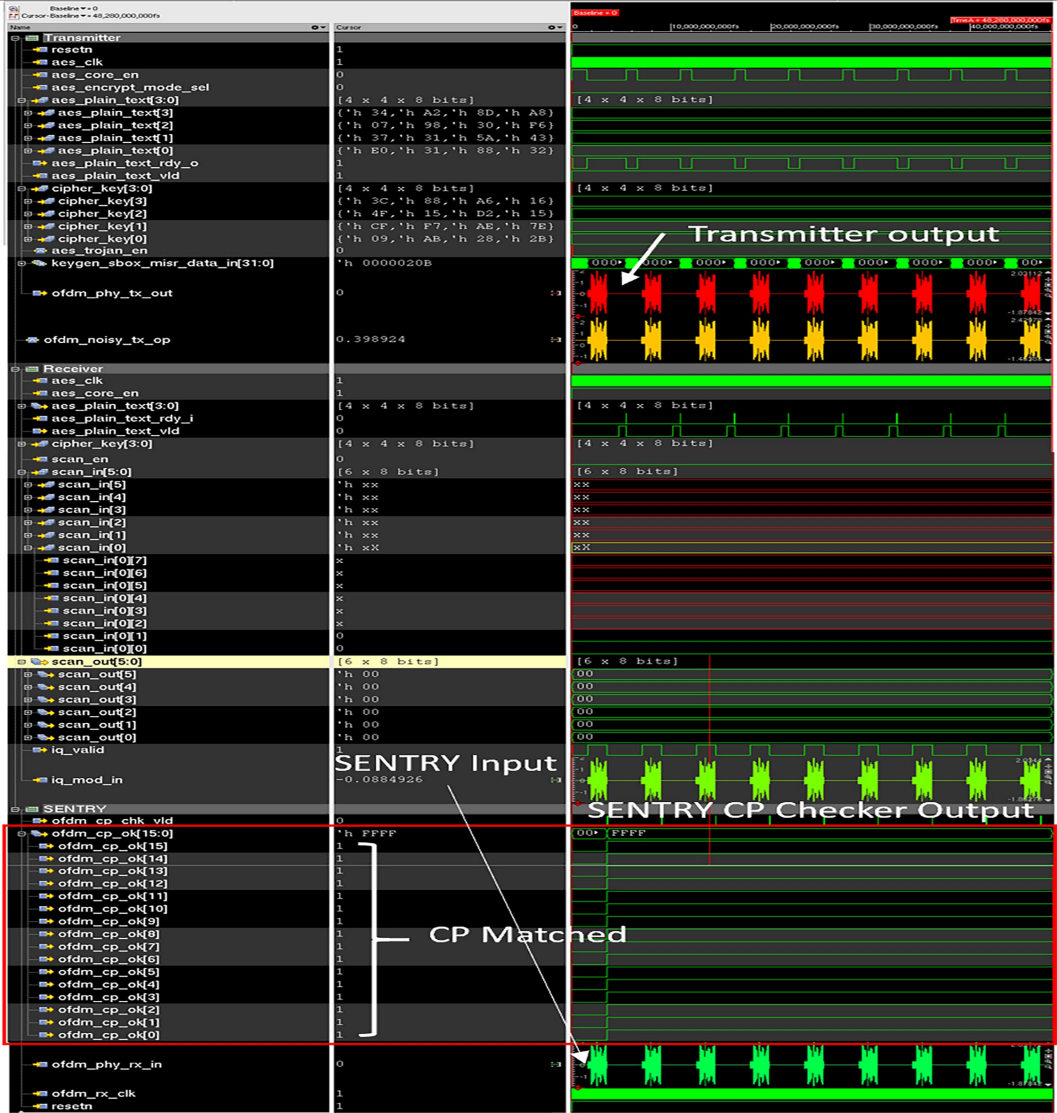
Simulation waveform of SENTRY with trojan free wireless cryptographic IC.

As proposed trojan will remain dormant during pre-silicon and post-silicon testing, so a continuous check after deployment is needed to protect the data security. The proposed HT detection mechanism uses an on-chip checker named “SENTRY” that acts as a CP checker to validate the transmitted CP at the transmitter end using minimum processing power and notify the sender if the CP becomes invalid in the transmission end to identify suspicious activity. Fig 18 shows the simulation waveform of SENTRY with Trojan Free circuit. As shown in Fig 18 the “ofdm_cp_chk_vld” signal is high whenever a check is performed in the transmitted OFDM waveform. In this case, a total of nine CP checks are performed for the nine transmitted OFDM symbols. “ofdm_cp_ok [15:0]” bus shows the results of the CP check. Bit 0 to Bit 7 of the “ofdm_cp_ok” bus is set to high if data 0 to data 7 matches with data 47 of the OFDM symbol and low otherwise. 8^th^ bit of the “ofdm_cp_ok” is set to high if data location 8 of the OFDM symbol matches with data location 46 of the OFDM symbol. The 9^th^ bit of the “ofdm_cp_ok” is set to high if data location 9 matches with data location 45 and is set to low otherwise. The rest of the bits 10–15 of the “ofdm_cp_ok” bus is set using the same principle. In a trojan-free case all sixteen data points of the ECP data matches with their corresponding data location in the OFDM data payload. Hence, all bits of “ofdm_cp_ok” is set to one resulting in a 16’hFFFF value.

**Fig 19:**
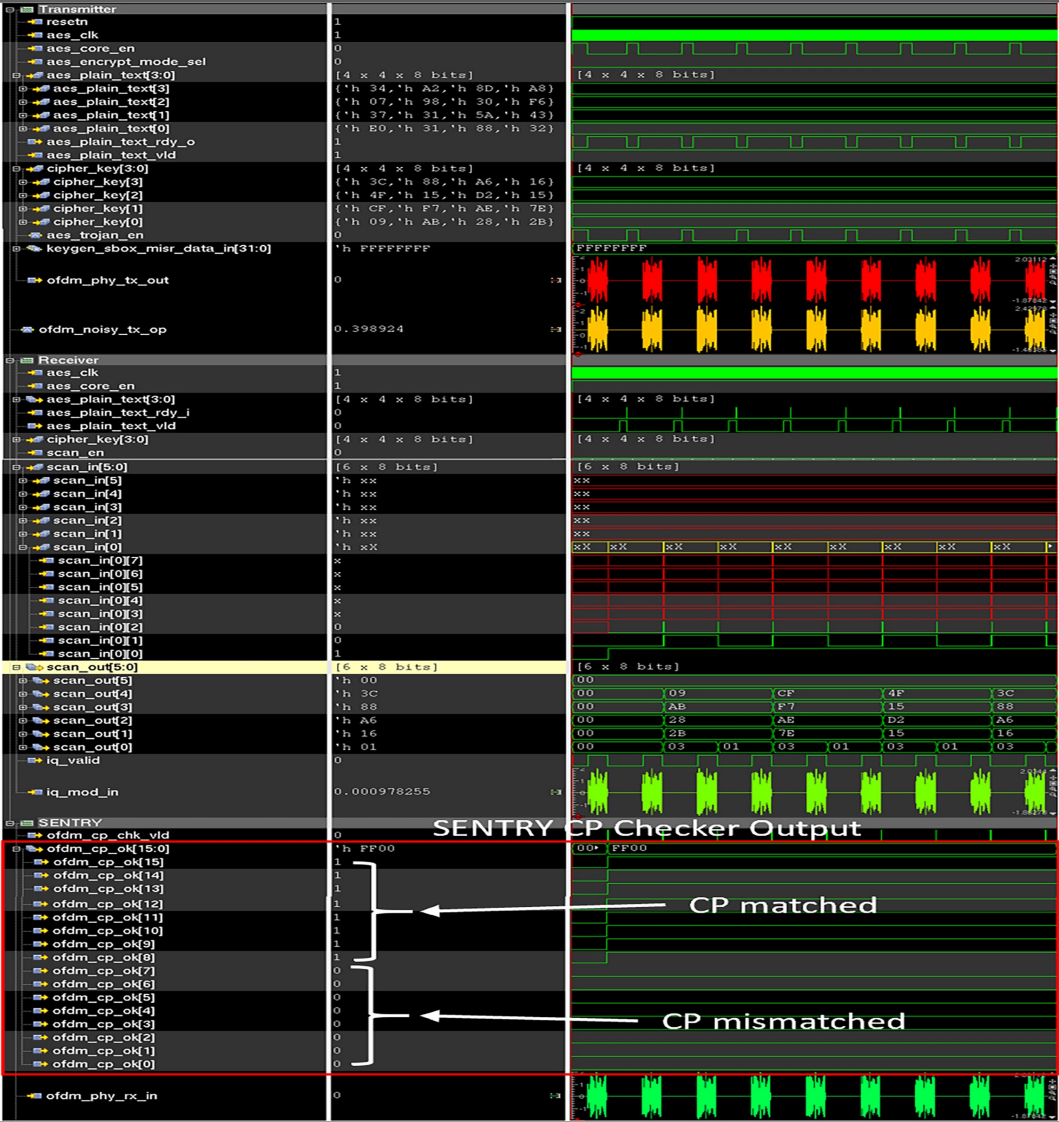
Simulation waveform of SENTRY with trojan infested wireless crypto IC.

In the case of trojan-infested circuit data location, 0 to 7 of the OFDM symbol is used for trojan data insertion. So, as shown in Fig 19, bit 0 to 7 of the “ofdm_cp_ok” bus is set to 0 because of data mismatches in these locations. Hence, the bus contains 16’hFF00 indicating the suspicious transmitter behavior.

## Discussion on results

By analyzing the functional simulation results it is observed that during the dormant stage HT infested ICs are indistinguishable from HT free ICs. After triggering, the HT requires at least 8 OFDM symbol transfers to completely leak the secret key to the attacker. As the CP is always discarded and never analyzed in the receiving end so, the legitimate users will be unaware of the effect of HT and by triggering the HT in the receiver the adversary can extract the embedded secret key. It is observed that a large value in the trojan activation counter increases the chance of detection avoidance during post-manufacturing testing. A drawback of the proposed HT model is that the transmitter HT trigger relies upon the presence of BIST circuitry to test the design. In absence of BIST circuitry, it will be difficult to implement the trigger circuit. But nowadays BIST is implemented in almost all wireless crypto IC for post-manufacturing tests.

To analyze the detection accuracy of the proposed HT detection mechanism SENTRY, 10 different data transmission scenarios are simulated using different plain text and cipher key matrix for HT-free ICs, and HT-infested ICs when HT is triggered. And it is found that SENTRY can detect the presence of HT in all 10 scenarios. Based on this observation it can be said that the proposed HT detection mechanism has 99.99% accuracy and does not suffer from any false-positive and false-negative results. The detection mechanism is also less computation-intensive and requires no previous data. However, a drawback of the proposed detection mechanism is that it increases the overall silicon area of the chip as SENTRY implementation requires around 10000 gates. However, this tradeoff is very advantageous compared to existing machine learning-based detection methods which require more computation circuits and silicon area compared to the proposed detection method.

### Comparison between proposed and existing hardware trojan threat models

Table 2 shows a comparison between the proposed Hardware Trojan threat model and previously implemented Hardware Trojan threat models.

**Table 2:** Comparison between proposed HT threat model and existing HT threat models.

Implementation Method Name	Trojan Type	Exploit Mechanism	Detection/ Prevention mechanism
Amplitude-Modulating Analog/RF Hardware Trojans in Wireless Networks [25]	Always On	Amplitude Modulation Offset Margin	information flow tracking-based proof-carrying hardware solution, Adaptive Channel Estimation Method to characterize transmission channel
Trojan Implementation on UWB Based Crypto IC [27–30]	Always On	Amplitude and Frequency Offset Margin	Statistical analysis of transmission power using PCA
INFECT on IEEE 802.11a/g [31]	Always On	FEC encoding of the transmitted signal	Monitoring the noise distribution at the receiver to identify systematic inconsistencies
RF Transmission Below Noise Level [32]	Always On	Transmitting rogue data below RF noise level using Spread-spectrum technique	Self-referencing method at transmitter using transmission noise profiling
Hardware Trojan Attacks in Analog/Mixed-Signal ICs via the Test Access Mechanism [8]	Trigger Based	Malicious scan in stimulus that is applied to analog IP	Password-based authentication and obfuscation of test circuits.
Proposed HT Threat Model	Trigger Based	ECP Property of OFDM	Analysis of CP at the transmission end using dedicated CP checker circuit

The key differences between the proposed HT method and previous methods are highlighted below,

- **Triggering mechanism:** Most of the previous research work like [27, 28, 31, 32] is based on “always-on” type HT. Hence, there is no dormant stage for previous implementations. The proposed implementation uses trigger-based HT. As during the dormant stage, HT infested ICs are indistinguishable from regular ICs so the proposed HT has a better chance of remaining undetected during the traditional post-manufacturing testing.
- **Payload:** Previous research works were focused on exploiting various properties of RF signals like amplitude, frequency [27–28], and delay spectrum [32]. The proposed HT threat model exploits the ECP properties of OFDM which is a novel exploit method.
- **Detection mechanism:** All previous research works like [27, 28, 31, 32] are reliant on machine learning-based statistical algorithms to characterize power and transmission channel noise distribution, which are very complex to implement. The proposed HT detection mechanism uses an on-chip CP checker named SENTRY which is a novel approach and simple to implement.
- **Run time checking:** Most of the previous research works were focused on detecting the presence of HT during post-manufacturing tests, not at runtime of the IC. This can be considered as a major drawback for detecting sequential HTs because due to large activation time-sequential HTs can remain dormant during the testing period thereby passing as good ICs. The proposed detection method uses a runtime on-chip CP checker, so it is effective for detecting sequential HTs.

## Conclusion

The development and implementation of a trigger-based HT for OFDM-based wireless cryptographic ICs that exploits the ECP property of OFDM protocol to embed trojan data inside valid OFDM waveform are presented in this paper. Simulation results show that to leak 128 bit AES cipher key the HT requires at least 8 OFDM symbol transfers. From the simulation result, it is also observed that during the dormant period it is impossible to distinguish between the HT free and HT infested ICs and a larger activation counter value increases the probability of passing Trojan Infested ICs as Trojan Free ICs for traditional post-silicon testing. The detection mechanism presented in this paper is an on-chip CP checker named SENTRY. It is fabricated separately using split fabrication techniques and assembled in a secure facility where the adversary can’t intervene. From the simulation, it is found that SENTRY can successfully distinguish between HT-free and HT-infested ICs when the HT is triggered. The proposed mechanism is compared with previous works and it is found that the proposed HT model has a greater chance of bypassing the traditional testing system. The future works may contain the development of statistical analysis methods to perform runtime analysis of power to detect the abnormal activities in the transmitter, development of preventive techniques against trojan insertion, and exploiting other parameters like constellation mapping property to develop trojan threat model.

## Supporting information

S1 Fig Block diagram of OFDM transmitter and receiver. (TIF)

S2 Fig Cyclic prefix property of OFDM communication scheme. (TIF)

S3 Fig System level block diagram of proposed wireless AES crypto IC. (TIF)

S4 Fig Detailed block diagram of trojan free IC. (TIF)

S5 Fig Flow chart of the AES based wireless transmitter module. (TIF)

S6 Fig Flowchart of the AES based wireless receiver module. (TIF)

S7 Fig Trojan trigger implementation by modifying LFSR generated using Leonardo spectrum. (TIF)

S8 Fig Trojan data insertion in ECP of an OFDM Symbol. (TIF)

S9 Fig Flowchart of HT infested wireless transmitter module. (TIF)

S10 Fig Detailed block diagram of trojan infested IC. (TIF)

S11 Fig Flowchart of HT infested wireless receiver module. (TIF)

S12 Fig Placement of SENTRY in detailed block diagram of cryptographic IC. (TIF)

S13 Fig Detailed block diagram of SENTRY. (TIF)

S14 Fig Flowchart of SENTRY. (TIF)

S15 Fig Simulation waveform of trojan free wireless cryptographic IC. (TIF)

S16 Fig Simulation waveform of trojan infested wireless cryptographic IC when HT is dormant. (TIF)

S17 Fig Simulation waveform of the trojan infested wireless cryptographic IC when HT is active. (TIF)

S18 Fig Simulation waveform of SENTRY with trojan free wireless cryptographic IC. (TIF)

S19 Fig Simulation waveform of SENTRY with trojan infested wireless crypto IC. (TIF)

S1 Table Summarized threat model for AMS IC. (PDF)

S2 Table Comparison between proposed HT threat model and existing HT threat models. (PDF)

S1 File System verilog codes repository for HT infested IC and sentry. (RAR)

## References

[1] McClean B, The McClean report 2018, 2017, section 3. leading IC suppliers and foundries, Available at:s, [Accessed 1 January 2018].

[2] IC Insight, 2017. Research Bulletin. [online] IC Insight, pp.1-3. Available at: http://www.icinsights.com/data/articles/documents/945.pdf [Accessed 1 November 2017].

[3] Force T. High performance microchip supply. Annual Report. Defense Technical Information Center (DTIC), USA. 2005 Feb.

[4] Guin U, DiMase D, Tehranipoor M. Counterfeit integrated circuits: Detection, avoidance, and the challenges ahead. Journal of Electronic Testing. 2014 Feb 1;30(1):9–23.

[5] Liu B, Qu G. VLSI supply chain security risks and mitigation techniques: A survey. Integration. 2016 Sep 1;55:438–48.

[6] Mishra P, Bhunia S, Tehranipoor M, editors. Hardware IP security and trust. Springer International Publishing; 2017 Jan 3.

[7] Tehranipoor M, Koushanfar F. A survey of hardware trojan taxonomy and detection. IEEE design & test of computers. 2010 Feb 5;27(1):10–25.

[8] Elshamy M, Di Natale G, Pavlidis A, Louërat MM, Stratigopoulos HG. Hardware Trojan Attacks in Analog/Mixed-Signal ICs via the Test Access Mechanism. InIEEE European Test Symposium 2020 May 25.

[9] Xiao K, Forte D, Jin Y, Karri R, Bhunia S, Tehranipoor M. Hardware trojans: Lessons learned after one decade of research. ACM Transactions on Design Automation of Electronic Systems (TODAES). 2016 May 27;22(1):1–23. doi: 10.1145/2906147

[10] Adee S. The hunt for the kill switch. iEEE SpEctrum. 2008 May 2;45(5):34–9.

[11] Skorobogatov S, Woods C. Breakthrough silicon scanning discovers backdoor in military chip. InInternational Workshop on Cryptographic Hardware and Embedded Systems 2012 Sep 9 (pp. 23–40). Springer, Berlin, Heidelberg.

[12] Bhunia S, Hsiao MS, Banga M, Narasimhan S. Hardware Trojan attacks: Threat analysis and countermeasures. Proceedings of the IEEE. 2014 Jul 15;102(8):1229–47. doi: 10.1109/JPROC.2014.2334493

[13] Lin L, Burleson W, Paar C. MOLES: malicious off-chip leakage enabled by side-channels. In2009 IEEE/ACM International Conference on Computer-Aided Design-Digest of Technical Papers 2009 Nov 2 (pp. 117–122). IEEE.

[14] Karri R, Rajendran J, Rosenfeld K, Tehranipoor M. Trustworthy hardware: Identifying and classifying hardware trojans. Computer. 2010 Oct 14;43(10):39–46.

[15] Rad R, Plusquellic J, Tehranipoor M. Sensitivity analysis to hardware Trojans using power supply transient signals. In2008 IEEE International Workshop on Hardware-Oriented Security and Trust 2008 Jun 9 (pp. 3–7). IEEE.

[16] Rad RM, Wang X, Tehranipoor M, Plusquellic J. Power supply signal calibration techniques for improving detection resolution to hardware Trojans. In2008 IEEE/ACM International Conference on Computer-Aided Design 2008 Nov 10 (pp. 632–639). IEEE.

[17] Jin Y, Makris Y. Hardware Trojan detection using path delay fingerprint. In2008 IEEE International workshop on hardware-oriented security and trust 2008 Jun 9 (pp. 51–57). IEEE.

[18] Du D, Narasimhan S, Chakraborty RS, Bhunia S. Self-referencing: A scalable side-channel approach for hardware Trojan detection. In International Workshop on Cryptographic Hardware and Embedded Systems 2010 Aug 17 (pp. 173–187). Springer, Berlin, Heidelberg.

[19] Yin CE, Qu G. Temperature-aware cooperative ring oscillator PUF. In2009 IEEE International Workshop on Hardware-Oriented Security and Trust 2009 Jul 27 (pp. 36–42). IEEE.

[20] Merli D, Heyszl J, Heinz B, Schuster D, Stumpf F, Sigl G. Localized electromagnetic analysis of RO PUFs. In2013 IEEE International Symposium on Hardware-Oriented Security and Trust (HOST) 2013 Jun 2 (pp. 19–24). IEEE.

[21] Antonopoulos A, Kapatsori C, Makris Y. Trusted analog/mixed-signal/RF ICs: A survey and a perspective. IEEE Design & Test. 2017 Jul 18;34(6):63–76.

[22] Polian I. Security aspects of analog and mixed-signal circuits. In2016 IEEE 21st International Mixed-Signal Testing Workshop (IMSTW) 2016 Jul 4 (pp. 1–6). IEEE.

[23] Antonopoulos A, Kapatsori C, Makris Y. Hardware Trojans in analog, mixed-signal, and RF ICs. InThe Hardware Trojan War 2018 (pp. 101–123). Springer, Cham.

[24] Subramani K, Volanis G, Bidmeshki MM, Antonopoulos A, Makris Y. Trusted and Secure Design of Analog/RF ICs: Recent Developments. In2019 IEEE 25th International Symposium on On-Line Testing and Robust System Design (IOLTS) 2019 Jul 1 (pp. 125–128). IEEE.

[25] Subramani KS, Helal N, Antonopoulos A, Nosratinia A, Makris Y. Amplitude-Modulating Analog/RF Hardware Trojans in Wireless Networks: Risks and Remedies. IEEE Transactions on Information Forensics and Security. 2020 Apr 27.

[26] Subramani KS, Antonopoulos A, Abotabl AA, Nosratinia A, Makris Y. ACE: Adaptive channel estimation for detecting analog/RF trojans in WLAN transceivers. In2017 IEEE/ACM International Conference on Computer-Aided Design (ICCAD) 2017 Nov 13 (pp. 722–727). IEEE.

[27] Liu Y, Jin Y, Makris Y. Hardware Trojans in wireless cryptographic ICs: silicon demonstration & detection method evaluation. In2013 IEEE/ACM International Conference on Computer-Aided Design (ICCAD) 2013 Nov 18 (pp. 399–404). IEEE.

[28] Liu Y, Jin Y, Nosratinia A, Makris Y. Silicon demonstration of hardware Trojan design and detection in wireless cryptographic ICs. IEEE Transactions on Very Large Scale Integration (VLSI) Systems. 2016 Dec 21;25(4):1506–19.

[29] Liu Y, Huang K, Makris Y. Hardware Trojan detection through golden chip-free statistical side-channel fingerprinting. InProceedings of the 51st Annual Design Automation Conference 2014 Jun 1 (pp. 1–6).

[30] Liu Y, Volanis G, Huang K, Makris Y. Concurrent hardware Trojan detection in wireless cryptographic ICs. In2015 IEEE International Test Conference (ITC) 2015 Oct 6 (pp. 1–8). IEEE.

[31] Subramani KS, Antonopoulos A, Abotabl AA, Nosratinia A, Makris Y. INFECT: INconspicuous FEC-based Trojan: A hardware attack on an 802.11 a/g wireless network. In2017 IEEE International Symposium on Hardware Oriented Security and Trust (HOST) 2017 May 1 (pp. 90–94). IEEE.

[32] Chang D, Bakkaloglu B, Ozev S. Enabling unauthorized RF transmission below noise floor with no detectable impact on primary communication performance. In2015 IEEE 33rd VLSI Test Symposium (VTS) 2015 Apr 27 (pp. 1–4). IEEE.

[33] Kapatsori C, Liu Y, Antonopoulos A, Makris Y. Hardware Dithering: A Run-Time Method for Trojan Neutralization in Wireless Cryptographic ICs. In2018 IEEE International Test Conference (ITC) 2018 (pp. 1–7). IEEE.

[34] Bidmeshki MM, Antonopoulos A, Makris Y. Information flow tracking in analog/mixed-signal designs through proof-carrying hardware IP. InDesign, Automation & Test in Europe Conference & Exhibition (DATE), 2017 2017 Mar 27 (pp. 1703–1708). IEEE.

[35] Karabacak F, Ogras UY, Ozev S. Detection of malicious hardware components in mobile platforms. In2016 17th International Symposium on Quality Electronic Design (ISQED) 2016 Mar 15 (pp. 179–184). IEEE.

[36] Karabacak F, Ogras U, Ozev S. Remote detection of unauthorized activity via spectral analysis. ACM Transactions on Design Automation of Electronic Systems (TODAES). 2018 Nov 28;23(6):1–21.

[37] Farshad Ali L. Harware Trojan for OFDM based wireless Cryptographic ICs. Proceedings of International Confernec on Informatics, Electronics & Vision, Kyoto, Japan 2020

[38] Ahmadi S. LTE-Advanced: a practical systems approach to understanding 3GPP LTE releases 10 and 11 radio access technologies. Academic Press; 2013 Oct 10.

