# Supplementary figures and images for: Analog hardware trojan design and detection in OFDM based wireless cryptographic ICs
Source: PLoS One. 2021 Jul 29;16(7):e0254903. doi: 10.1371/journal.pone.0254903 (PMC8321131; doi:10.1371/journal.pone.0254903)

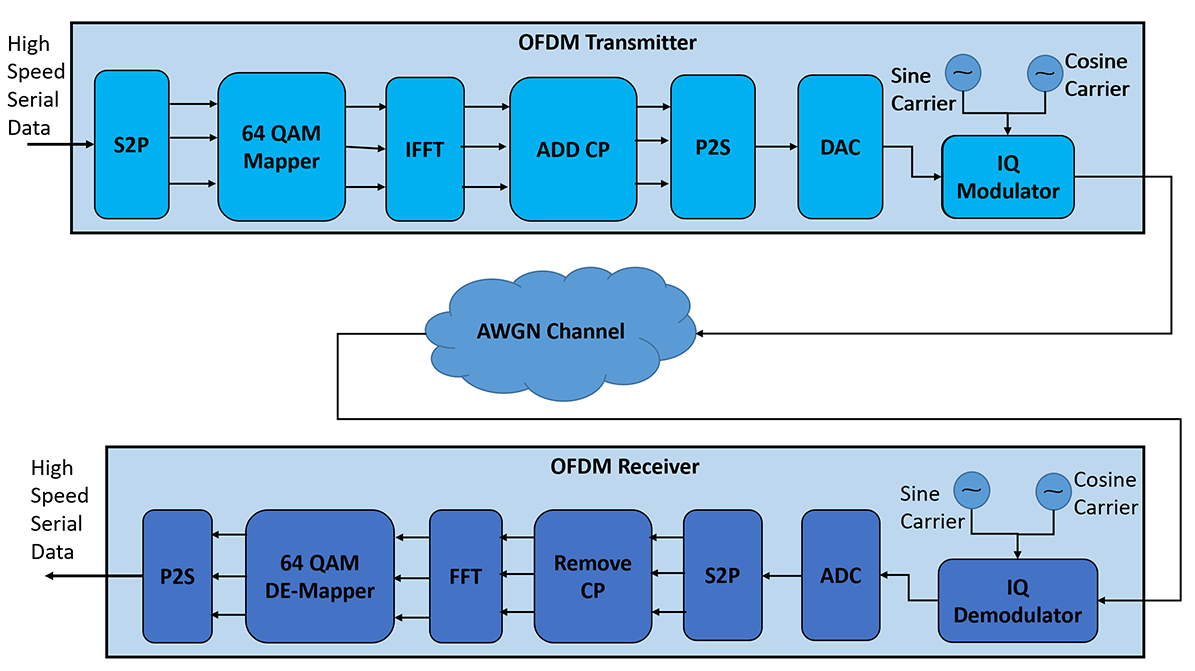

Supplement: S1 Fig — (TIF) [file pone.0254903.s001.tif]

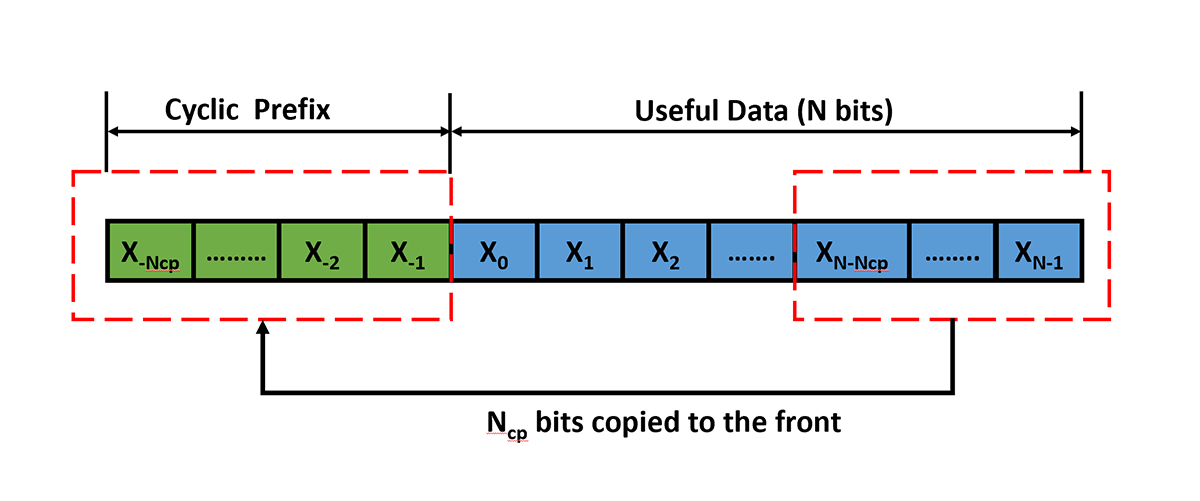

Supplement: S2 Fig — (TIF) [file pone.0254903.s002.tif]

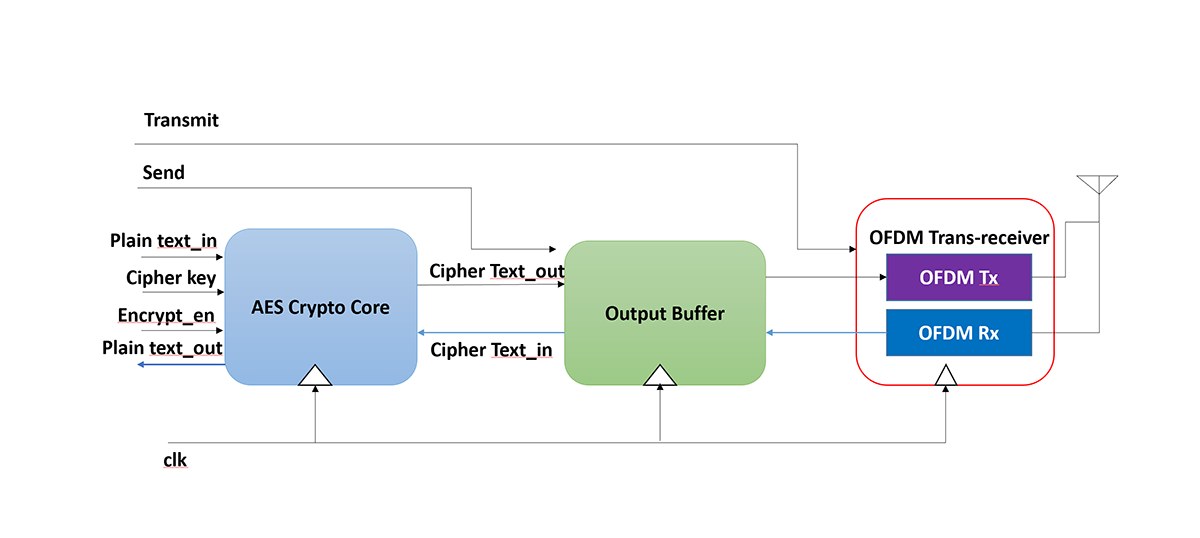

Supplement: S3 Fig — (TIF) [file pone.0254903.s003.tif]

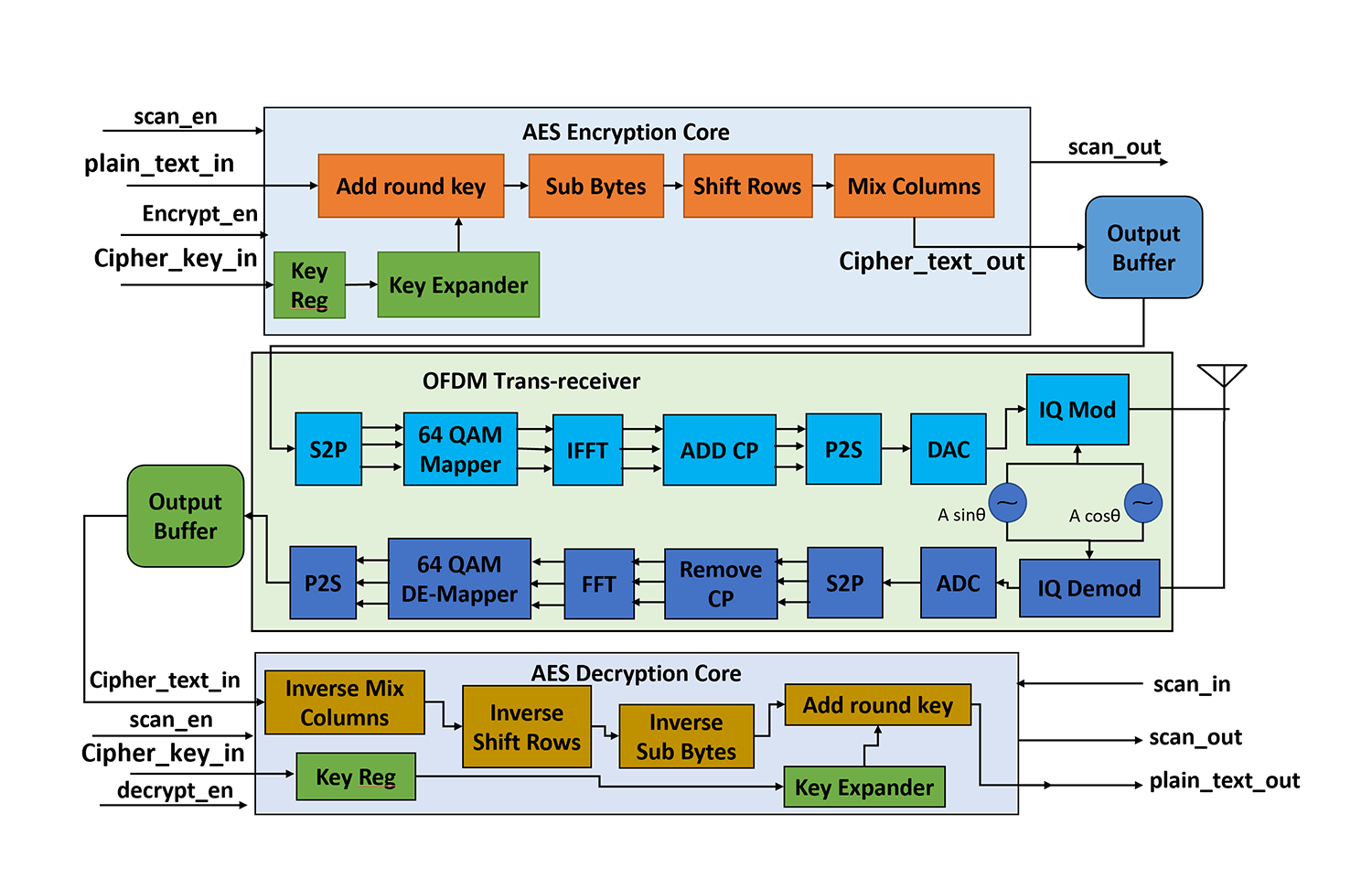

Supplement: S4 Fig — (TIF) [file pone.0254903.s004.tif]

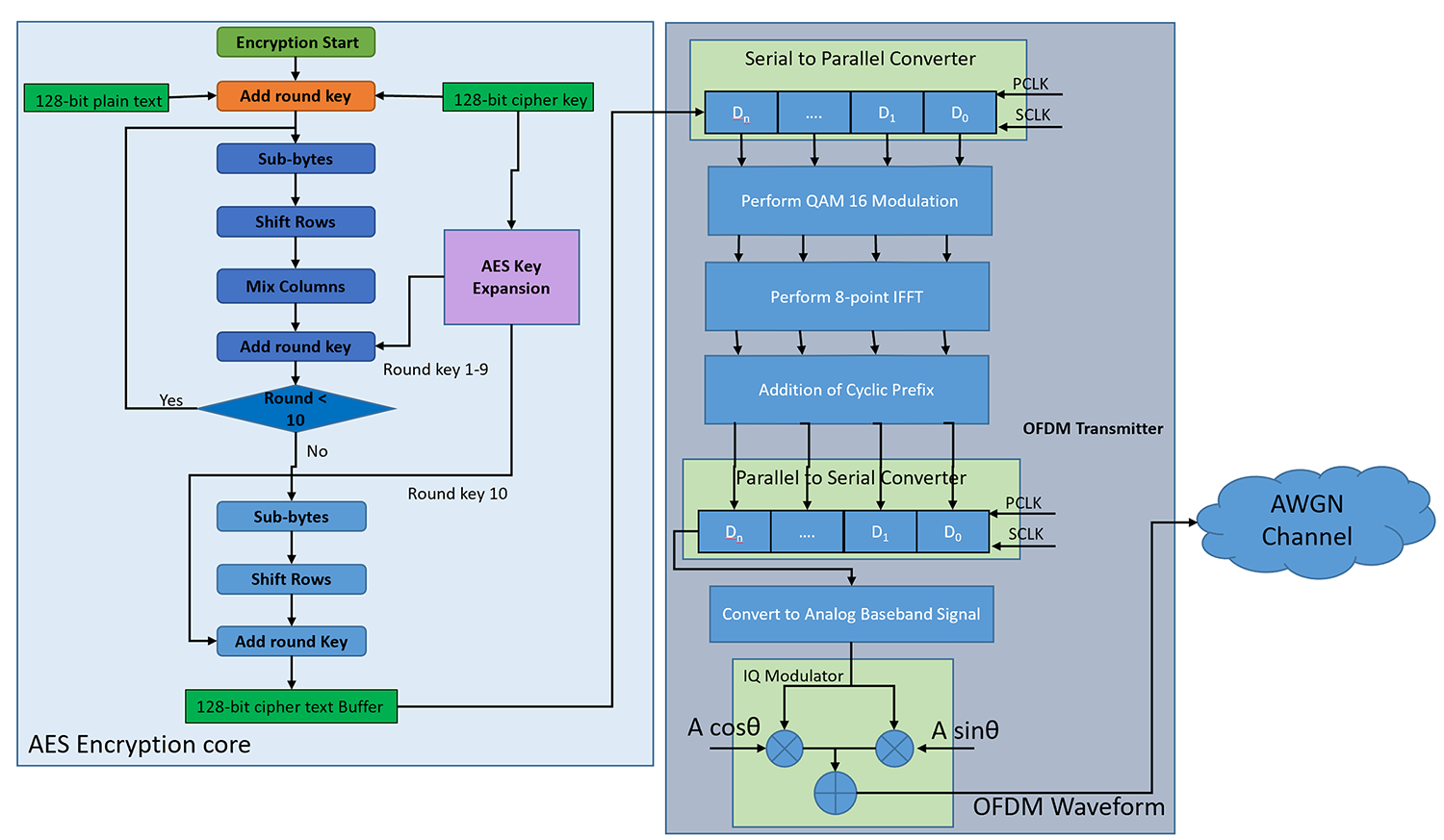

Supplement: S5 Fig — (TIF) [file pone.0254903.s005.tif]

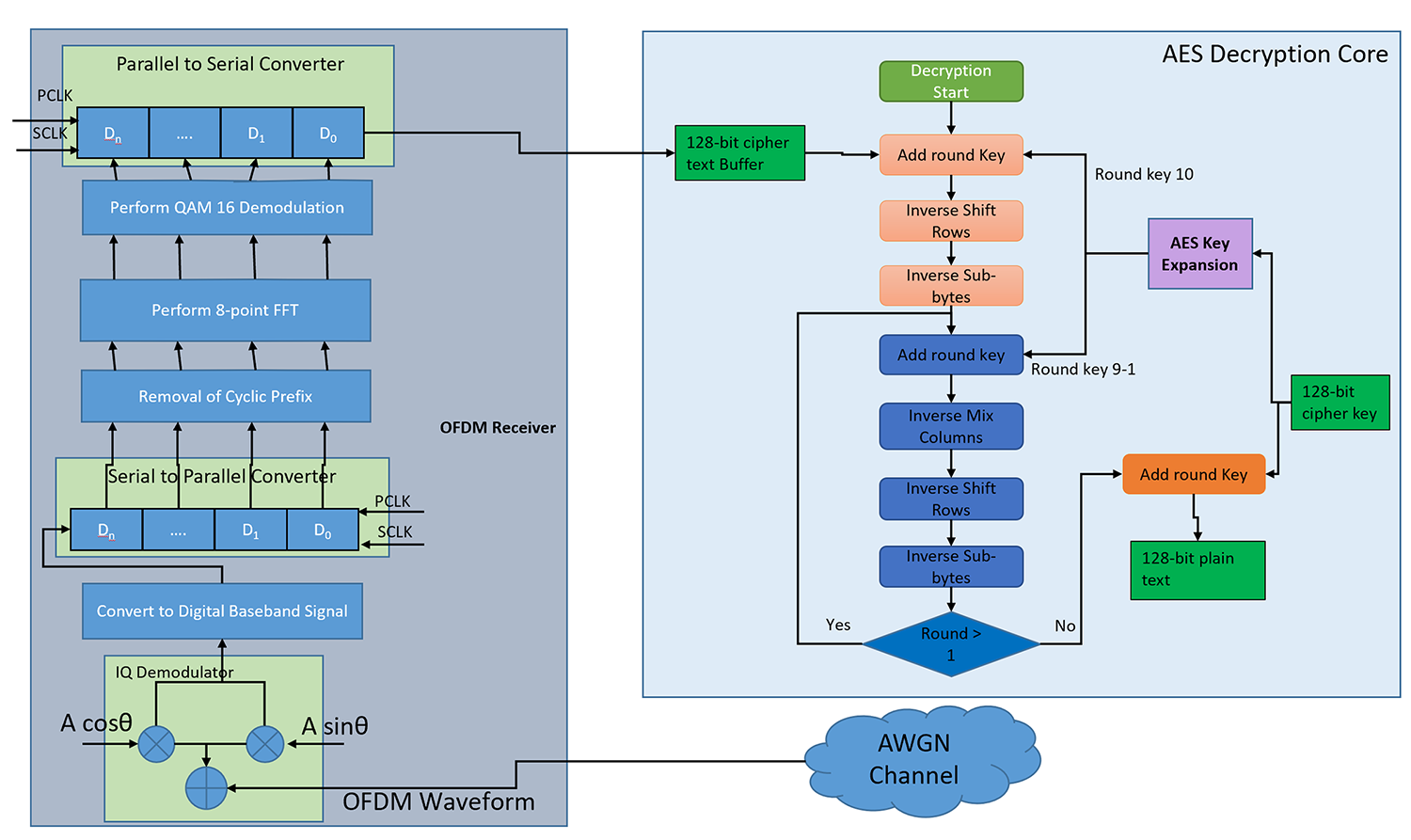

Supplement: S6 Fig — (TIF) [file pone.0254903.s006.tif]

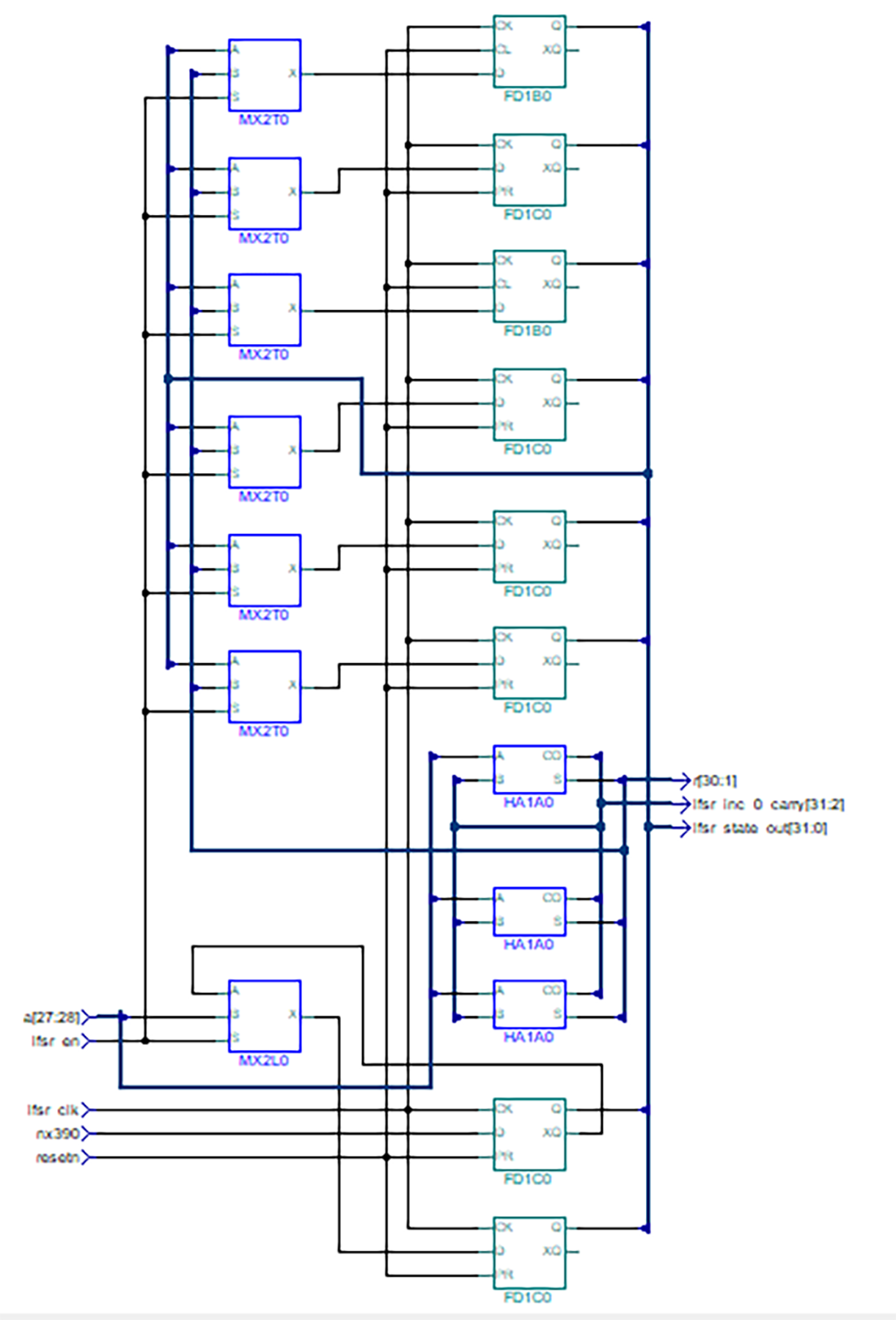

Supplement: S7 Fig — (TIF) [file pone.0254903.s007.tif]

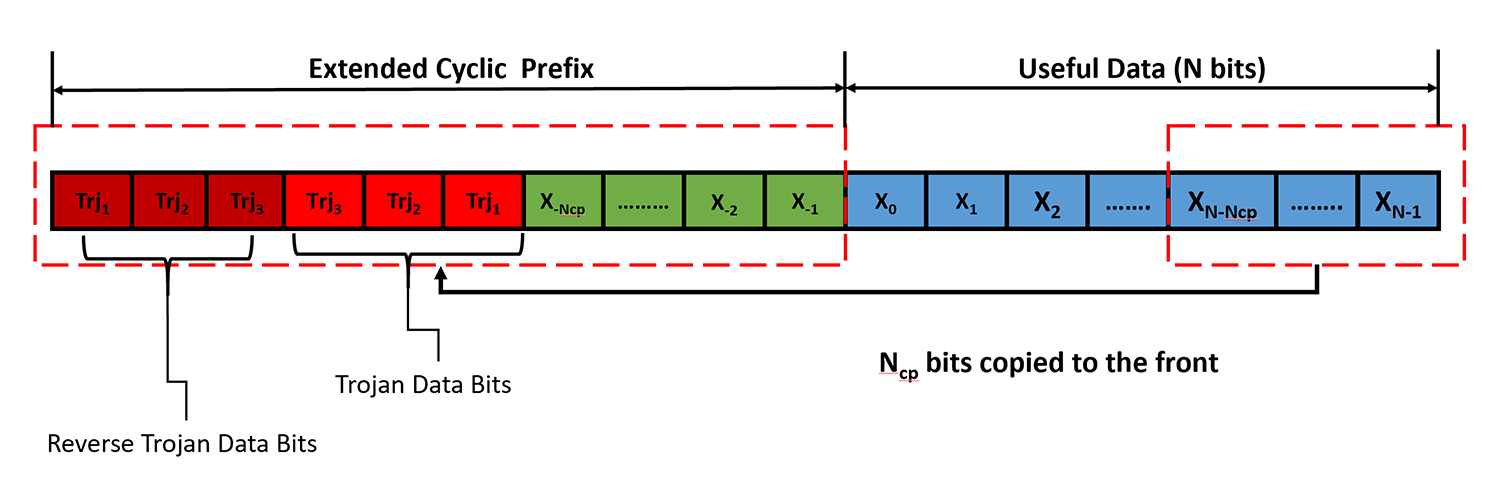

Supplement: S8 Fig — (TIF) [file pone.0254903.s008.tif]

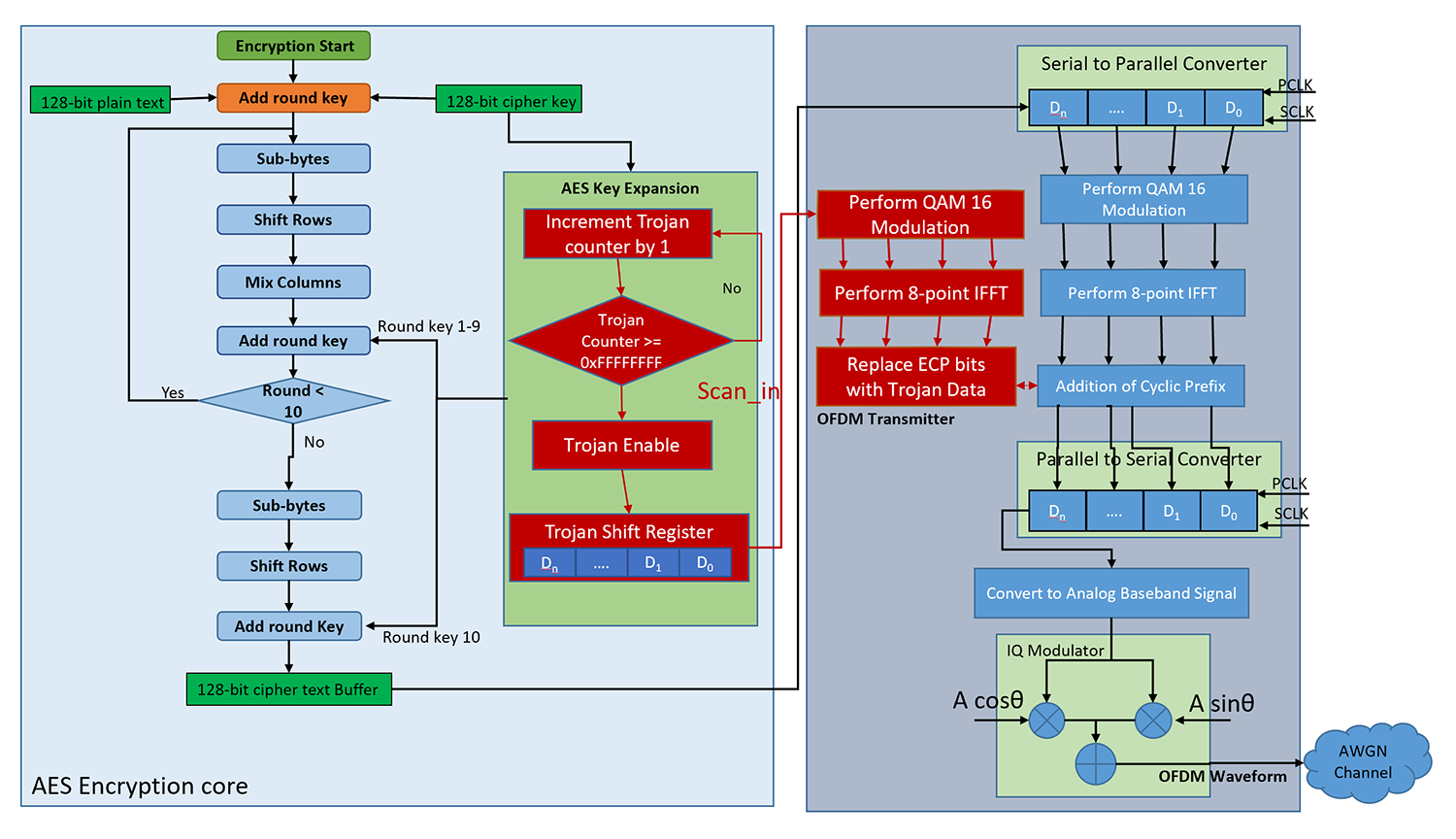

Supplement: S9 Fig — (TIF) [file pone.0254903.s009.tif]

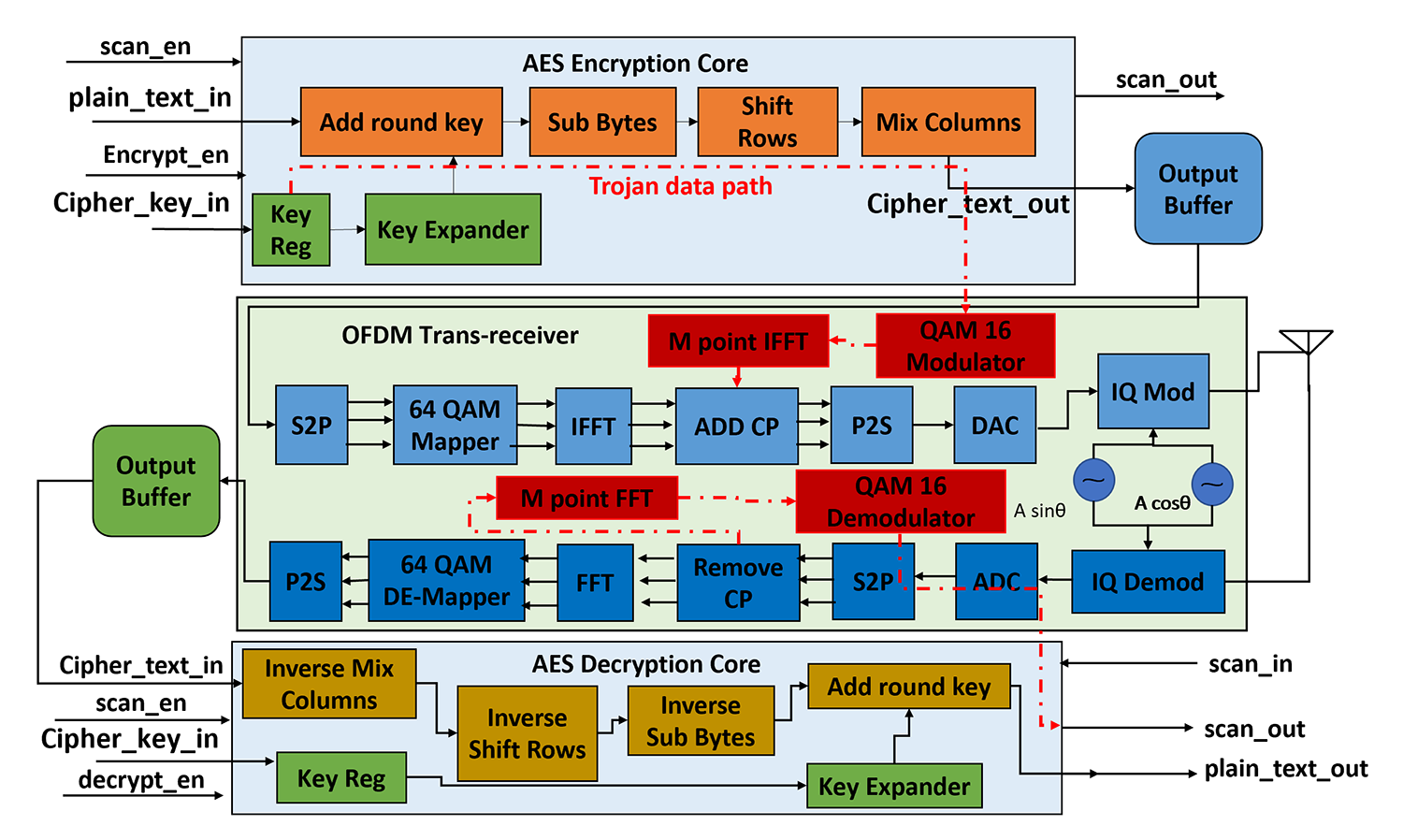

Supplement: S10 Fig — (TIF) [file pone.0254903.s010.tif]

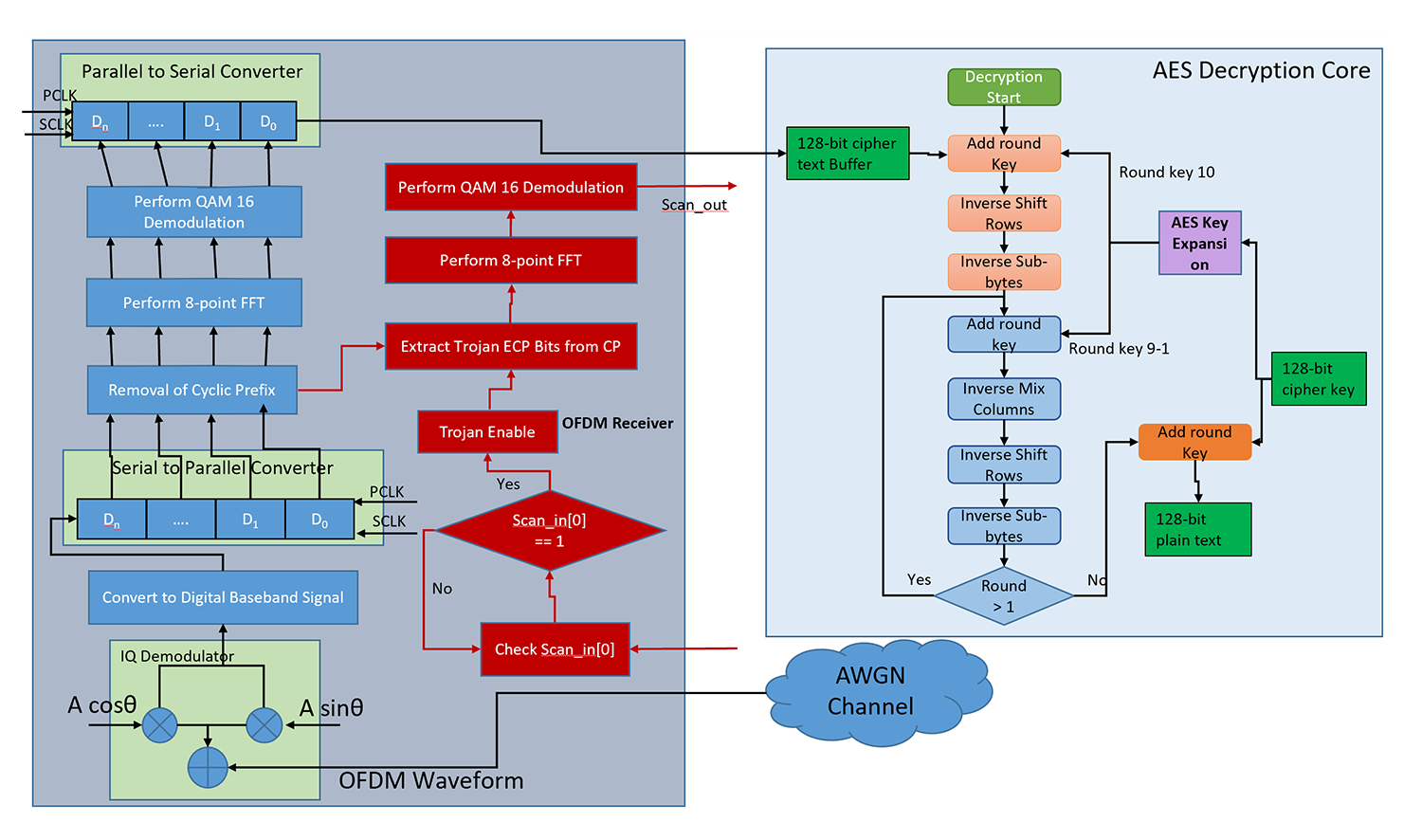

Supplement: S11 Fig — (TIF) [file pone.0254903.s011.tif]

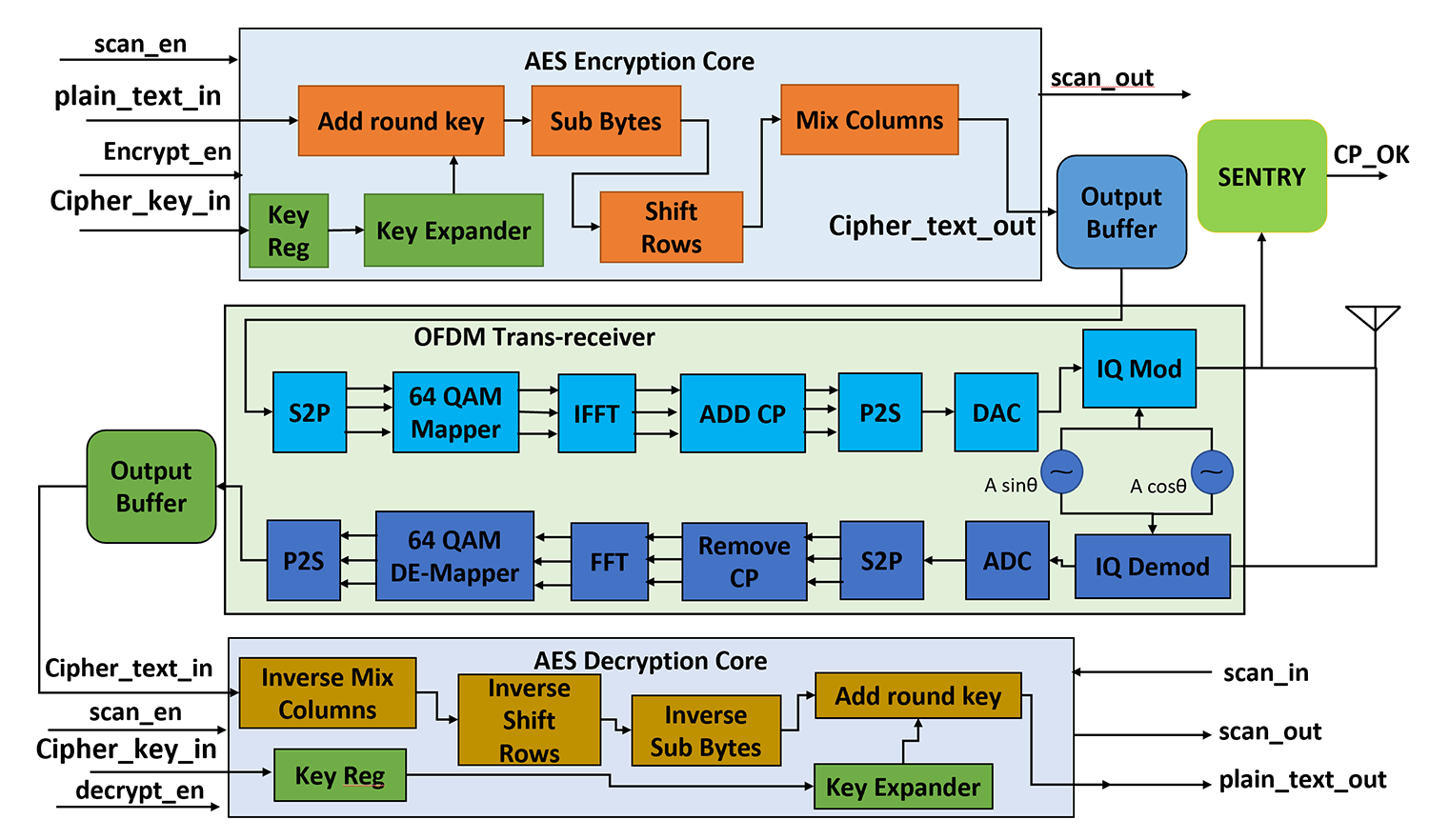

Supplement: S12 Fig — (TIF) [file pone.0254903.s012.tif]

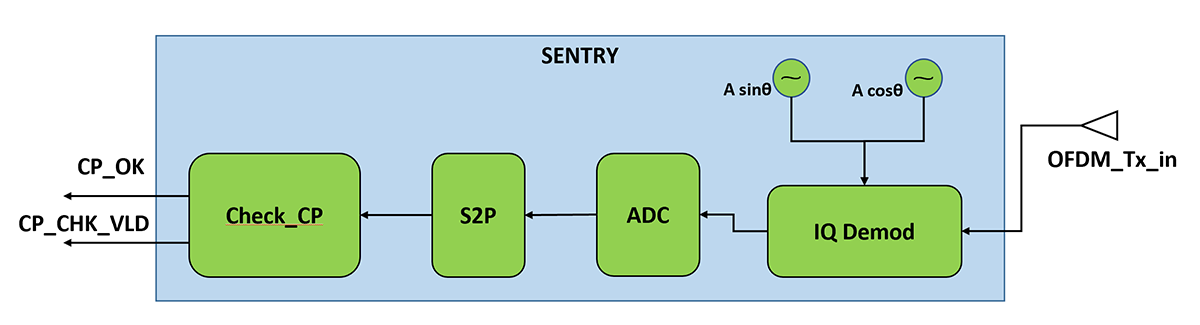

Supplement: S13 Fig — (TIF) [file pone.0254903.s013.tif]

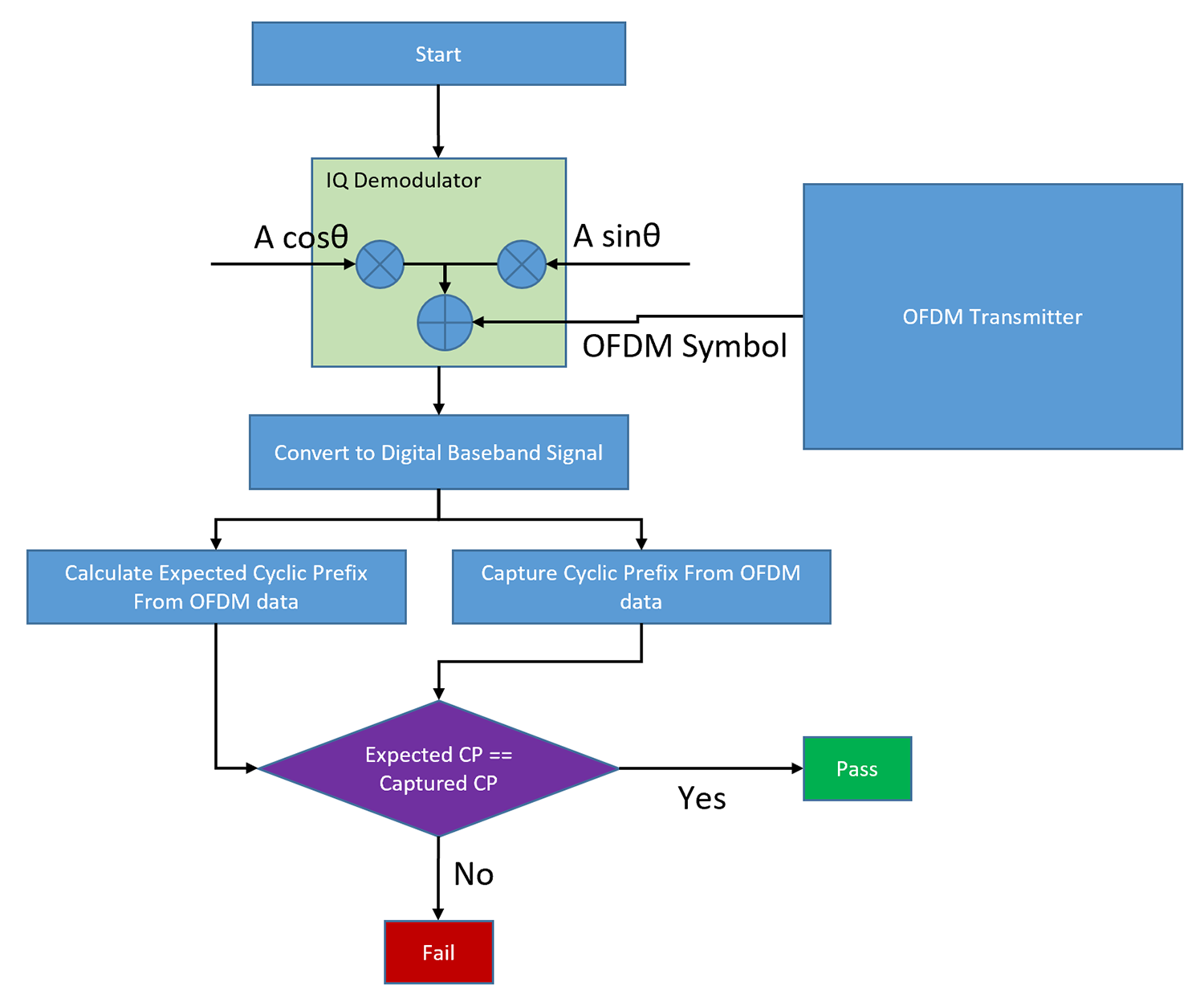

Supplement: S14 Fig — (TIF) [file pone.0254903.s014.tif]

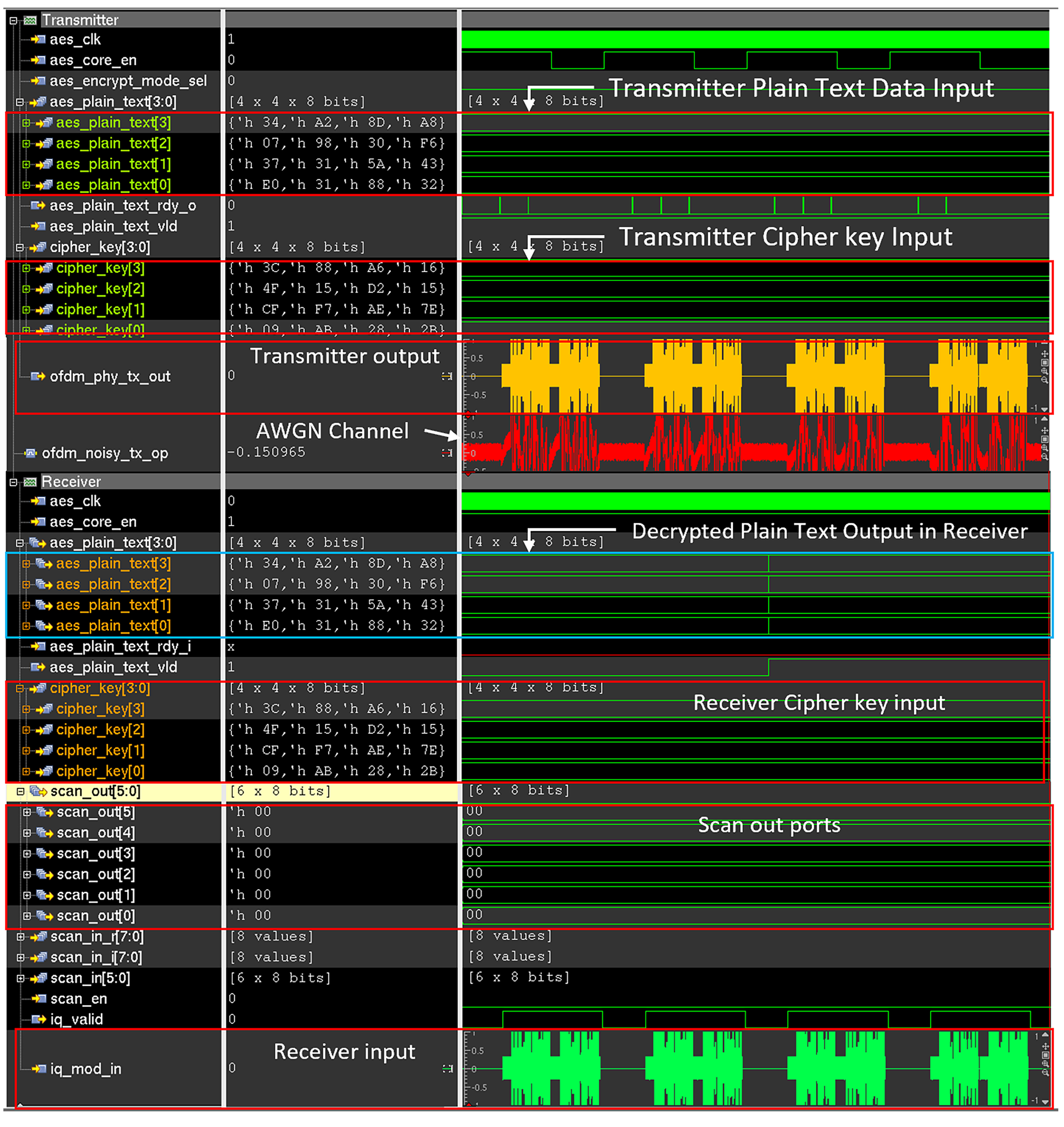

Supplement: S15 Fig — (TIF) [file pone.0254903.s015.tif]

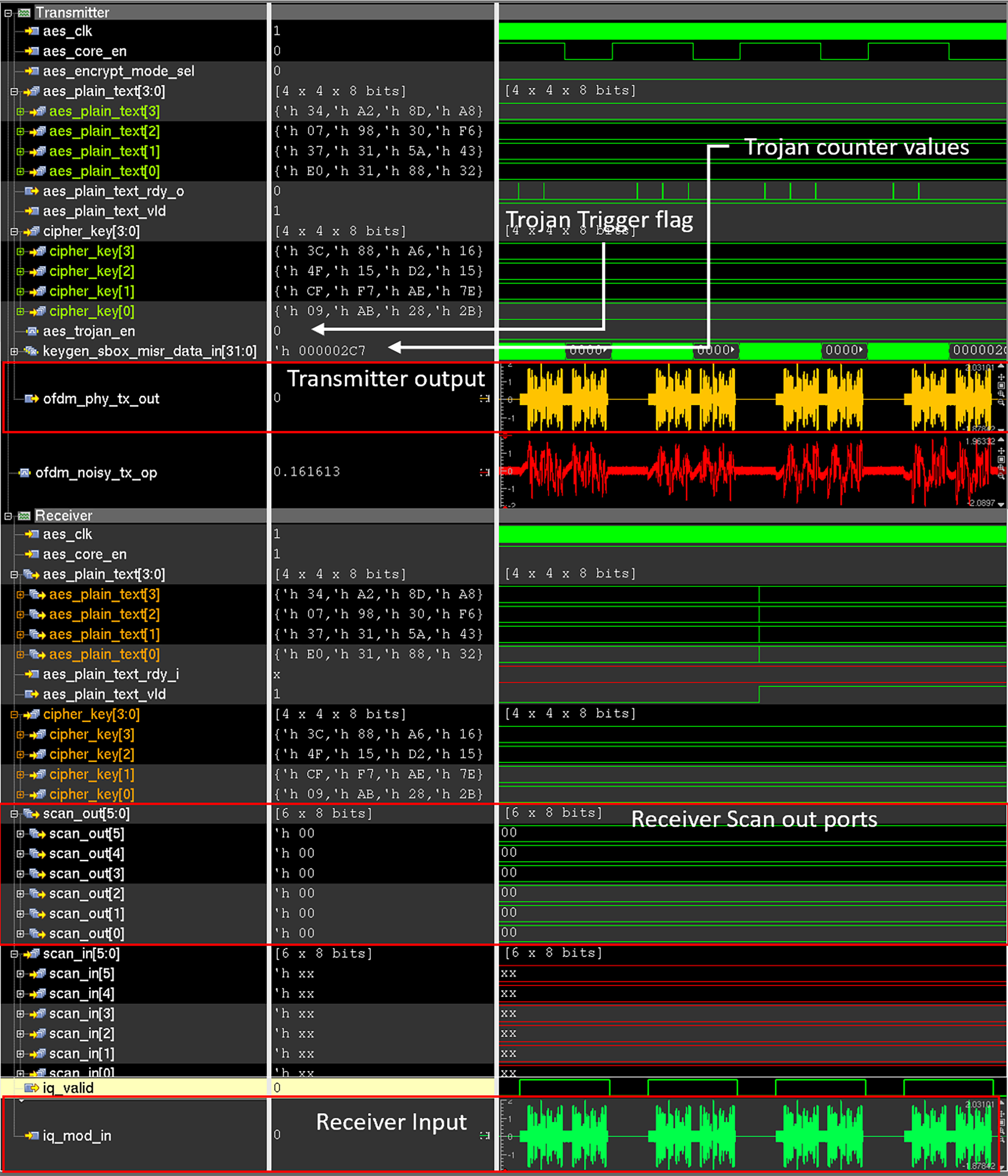

Supplement: S16 Fig — (TIF) [file pone.0254903.s016.tif]

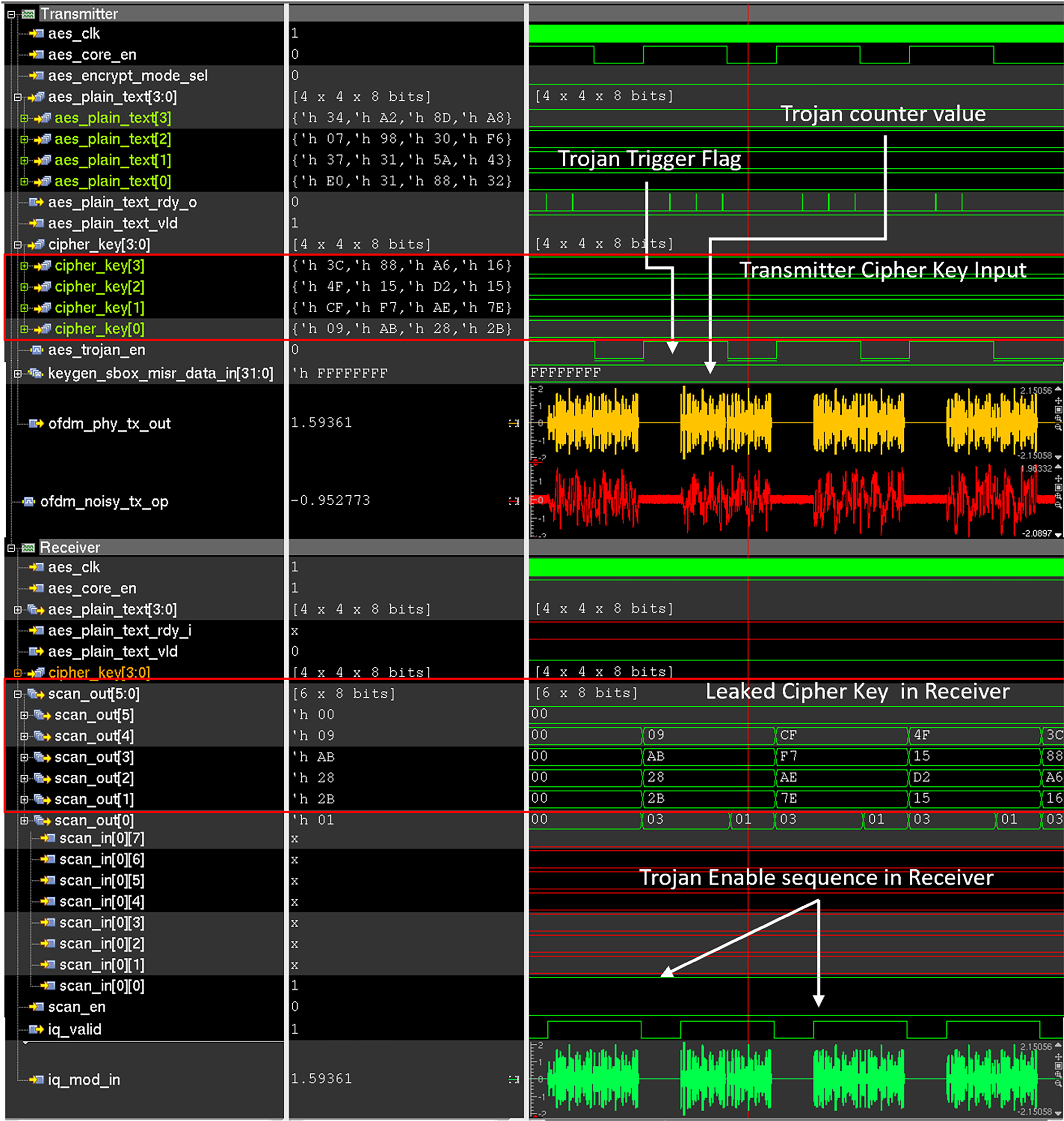

Supplement: S17 Fig — (TIF) [file pone.0254903.s017.tif]

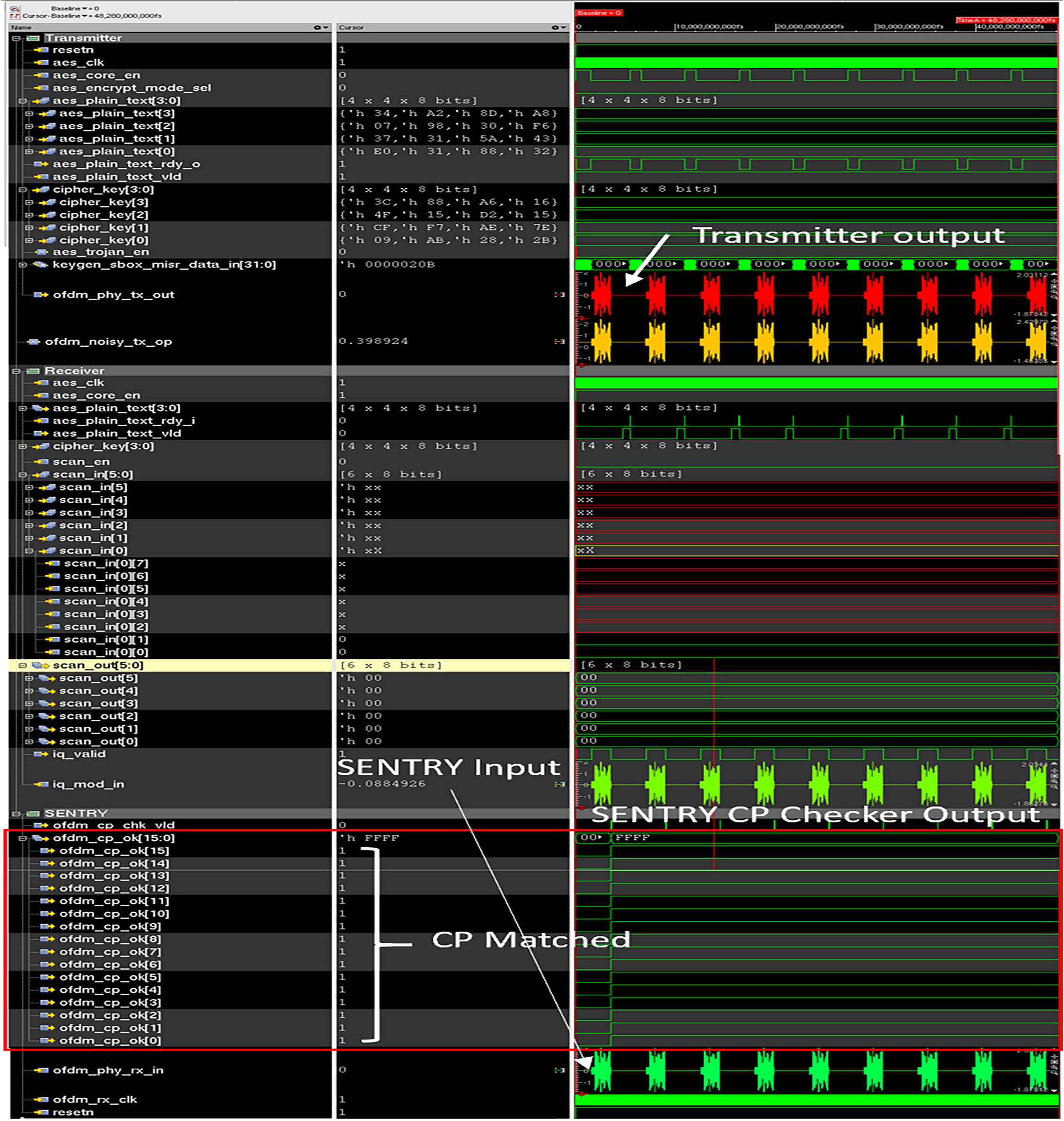

Supplement: S18 Fig — (TIF) [file pone.0254903.s018.tif]

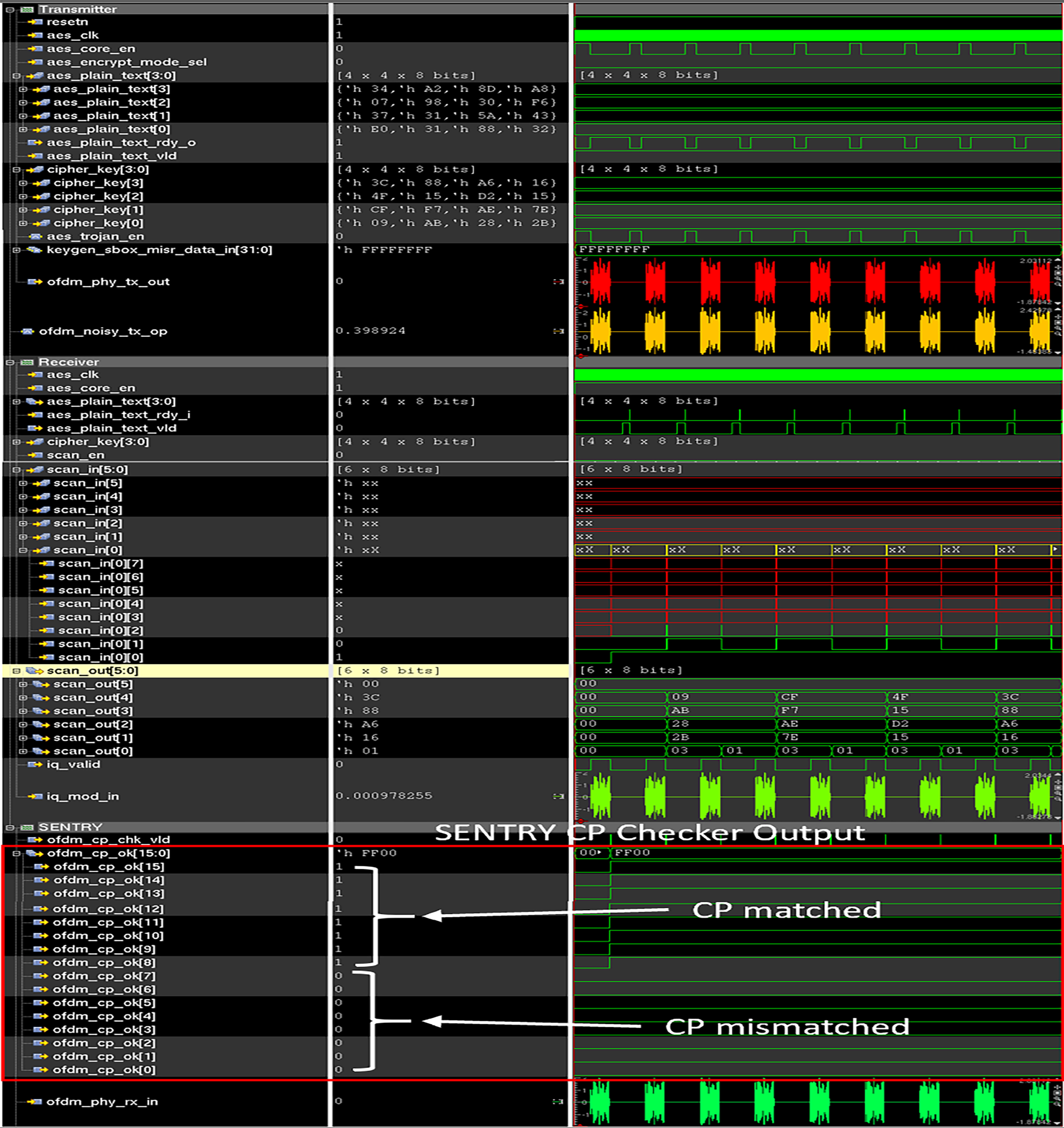

Supplement: S19 Fig — (TIF) [file pone.0254903.s019.tif]
