# Supplementary material for: Analog hardware trojan design and detection in OFDM based wireless cryptographic ICs
Source: PLoS One. 2021 Jul 29;16(7):e0254903. doi: 10.1371/journal.pone.0254903 (PMC8321131; doi:10.1371/journal.pone.0254903)
Supplement: S1 Table — (PDF) [file pone.0254903.s020.pdf]

HT Threat Model	Description	3PIP Vendor	Design Engineer	Foundry
**A**	Untrusted 3PIP Vendor	Untrusted	Trusted	Trusted
**B**	Untrusted EDA tool or Rouge Developer	Trusted	Untrusted	Trusted
**C**	Untrusted Design House	Untrusted	Untrusted	Trusted
**D**	Untrusted Foundry	Trusted	Trusted	Untrusted
**E**	Commercial off-the-shelf component	Untrusted	Untrusted	Untrusted
**F**	Fabless SoC design house	Untrusted	Trusted	Untrusted
