# Supplementary material for: Analog hardware trojan design and detection in OFDM based wireless cryptographic ICs
Source: PLoS One. 2021 Jul 29;16(7):e0254903. doi: 10.1371/journal.pone.0254903 (PMC8321131; doi:10.1371/journal.pone.0254903)
Supplement: S2 Table — (PDF) [file pone.0254903.s021.pdf]

**Implementation Method Name**	**Trojan Type**	**Exploit Mechanism**	**Detection/ Prevention mechanism**
Amplitude-Modulating Analog/RF Hardware Trojans in Wireless Networks [25]	Always On	Amplitude Modulation Offset Margin	information flow tracking-based proof-carrying hardware solution, Adaptive Channel Estimation Method to characterize transmission channel
Trojan Implementation on UWB Based Crypto IC [27-30]	Always On	Amplitude and Frequency Offset Margin	Statistical analysis of transmission power using PCA
INFECT on IEEE 802.11a/g [31]	Always On	FEC encoding of the transmitted signal	Monitoring the noise distribution at the receiver to identify systematic inconsistencies
RF Transmission Below Noise Level [32]	Always On	Transmitting rogue data below RF noise level using Spread-spectrum technique	Self-referencing method at transmitter using transmission noise profiling
Hardware Trojan Attacks in Analog/Mixed-Signal ICs via the Test Access Mechanism [8]	Trigger Based	Malicious scan in stimulus that is applied to analog IP	Password-based authentication and obfuscation of test circuits.
Proposed HT Threat Model	Trigger Based	ECP Property of OFDM	Analysis of CP at the transmission end using dedicated CP checker circuit
